# Supplementary material for: Molecular features of myosin F adapted for driving actin flows in Toxoplasma gondii
Source: J Cell Sci. 2026 Apr 17;139(7):jcs264520. doi: 10.1242/jcs.264520 (PMC13120683; doi:10.1242/jcs.264520)
Supplement: Supplementary information [file joces-139-264520-s1.pdf]

```
PVX_082400                  -----------------MDSVNKCVVGTKVFVRDKQKVWVCAEVVKEET--ELVVKTE--      39
PBANKA_1344100              -----------------MEPTSKCVVGTKIFIKHKEKVWISAEIIKEDT--DIVVKTD--      39
Py17XNL_001303339           -----------------MEPTNKCVVGTKIFIKHKEKVWISAEIIKEDT--DIVVKTD--      39
BOVATA_017340               ----------------MAEIASSMAVGTHLFVCSETEVWQEAVVDSISD-GKVFVRVKGA      43
EfaB_PLUS_276.g39           ------MAAGVLGASGPPEGEVRCPIGTRIYVPSPESVWATAAVVSVEANGAVVIKLDAE      54
Cvel_22959                  -----------------MASSDKYAVGTLVFVADEEMVWKPAEIIAVAGKE-ITVRTEGD      42
TgMyoF                      MTASSADGASAPGGGDPGEEEVRCAVGTKIYVPDAADVWRTAEVVKIQEDGSLTARVDAD      60
Ncaninum_LIV_000262900      MTASSLDGASPTGGGDQGDEEVRCAVGTKIYVSDPADVWKTAEVVKIQEDGSLTARVDAD      60
                                                     :** :::     **  * :        :  : .

PVX_082400                  -DDEIVVLKEKDEFHLKNLDVFDSNGLSAPADLTKLTHLHEASILHSLNVRFDIDEIYTF      98
PBANKA_1344100              -DDEIVNLKEGDEFFLRNTDIFNSNGLSAPPDLTKLTHLHEASVLHSLNIRFDIDEIYTF      98
Py17XNL_001303339           -DDEIVNLKEGDEFLLRNTDIFNSNGLSAPPDLTKLTHLHEASVLHSLNIRFDIDEIYTF      98
BOVATA_017340               QPGELVELKAGNKFHPHTEDRFNTP-SGYPDDLCNLTYLHEASVLHALDCRFAVDDIYTL      102
EfaB_PLUS_276.g39           -DRVI-TLKKGDPFYLCNTDEWNARGLCAPDDLTSLTHLHEAAVLDSLDIRFEVDAIYTF      112
Cvel_22959                  -QEERQVNSEKEPLYLRSAEIFSAEGLVGLDDLTQLMHLHEAALLDSLNTRFDVDKIYTF      101
TgMyoF                      -NELV-QLKKNDIWYLCNTDVWNTTGLSAPTDLTMLTHLHEAAVLDSLNLRFDIDEIYTF      118
Ncaninum_LIV_000262900      -NELV-HLRKGDLWYLCNTDVWNTTGLSAPTDLTMLTHLHEAAVLDSLNLRFDIDEIYTF      118
                                       :     . : :.:       **  * :****::*.:*: ** :* ***:

PVX_082400                  TGPILIAVNPFKMIPDLYSDSMLAKHVQ--------------------------------      126
PBANKA_1344100              TGPILIAINPFKNIKNLYSDNILEKHIQ--------------------------------      126
Py17XNL_001303339           TGPILIAINPFKHIKNLYSDNILEKHIQ--------------------------------      126
BOVATA_017340               TGKILIAVNPFKSIKGLYDDETIVRYLEM-------------------------------      131
EfaB_PLUS_276.g39           TGPILIAVNPFKTLRGLYDYSTLKRYILAHHASDVPPHHSTIEGTSRGGNSDGPSASPAG      172
Cvel_22959                  TGPILIAVNPFKTIPGLYEFDTLKSFIS--------------------------------      129
TgMyoF                      TGPILIAVNPFKQITGLYDMKQLVRYIASSELPMPGVPSSS--------S--GSSSNAPV      168
Ncaninum_LIV_000262900      TGPILIAVNPFKQIAGLYDMKQLVRYIASSQFPALPRPASS--------PSSTSAPSSPA      170
                            ** ****:**** : .**. . :  .:
```

Insert 1

```
PVX_082400                  -PIQSKSPHIFSTANSAYLGMCQHNKSQTILISGESGAGKTESTKYVMKFLACAGSD-IK      184
PBANKA_1344100              -PIKSKSPHIFATSNSAYLGMCKNNKSQTILISGESGAGKTESTKYVMKFLACAGSD-IK      184
Py17XNL_001303339           -PIKSKSPHIFATSNSAYLGMCKNNKSQTILISGESGAGKTESTKYVMKFLACAGSD-IK      184
BOVATA_017340               --CDHGVPHVFGVARDAYNNMTKNEQSQTILISGESGAGKTESTKFAMKFLAIAGAESME      189
EfaB_PLUS_276.g39           PRDILKEPHVFATSSAAYEGMCVHRRSQTILISGESGAGKTESTKFVMKFLACAGSDTLE      232
Cvel_22959                  -PKVSKTPHVFATANAAYRALCDTTRSQTVLISGESGAGKTESTKFVMKFLASAGTPDAT      188
TgMyoF                      ALPISRQPHVFASSSAAYQGMCNEKQSQTILISGESGAGKTESTKFVMKFLACAGSEDLE      228
Ncaninum_LIV_000262900      SLPISRQPHVFASSSAAYEGMCNEKQSQTILISGESGAGKTESTKFVMKFLACAGSDDLE      230
                                   **:*. :  **  :    :***:***************:.***** **:

PVX_082400                  RRSLIECQILESNPLLEAFGNARTLRNDNSSRFGKYIELQFSLDRKGGGQYYTRGKLCGA      244
PBANKA_1344100              KRSLIESQILESNPLLEAFGNSKTLRNNNSSRFGKYIELHFDICNNG----YIKGKLYGA      240
Py17XNL_001303339           KRSLIESQVLESNPLLEAFGNAKTLRNNNSSRFGKYIELHFDICNNG----YVKGKLYGA      240
BOVATA_017340               KKSPAEVKVLESNPLLESFGNASTVRNANSSRFGKFIELQYRKES------PIKARLVGA      243
EfaB_PLUS_276.g39           KRSTVEAQVLDSNPLLEAFGNARTLRNDNSSRFGKFIELQFFVAKDAH--GNPRGKLCGA      290
Cvel_22959                  SHSTVEKQVLESNPLLEAFGNARTLRNDNSSRFGKFIELQFKTEKDSE--GLVNGRVCGA      246
TgMyoF                      RRSQVEAQVLESNPLLEAFGNARTLRNDNSSRFGKFIELQFQTSKAKR-MSGNRGRLCGA      287
Ncaninum_LIV_000262900      RRSQVEAQVLESNPLLEAFGNARTLRNDNSSRFGKFIELQFQTNKTKR-VSGNRGRLCGA      289
                             :*   * ::*:******:***: *:** *******:***::            .:: **
```

Insert 2

```
PVX_082400                  KIRTYLLEKVRVCYQQEGERNYHIFYQLCRAAREAARGAANEAVSGEASEVVDEVVSESP      304
PBANKA_1344100              KILTYLLEKVRVCDQQEGERNYHIFYQLCSAVQYYKNKKLNL------------------      282
Py17XNL_001303339           KILTYLLEKVRVCDQQEGERNYHIFYQLCSAVQYYKNKKLNL------------------      282
BOVATA_017340               RIETYLLEKVRICQQQDGERNYHIFHQLTAAANRGE------------------------      279
EfaB_PLUS_276.g39           RIQTYLLEKVRVCQQQEGERNFHIFYQLCAAAARAE-QT---------------------      328
Cvel_22959                  RIQTYLLEKVRVVDQQEGERNFHIFYQLTAAFARGCKIE---------------------      285
TgMyoF                      RIQTYLLEKVRVCDQQEGERNYHIFYQLCAAAEAAA-QT---------------------      325
Ncaninum_LIV_000262900      RIQTYLLEKVRVCDQQEGERNYHIFYQLCAAAEAAA-QK---------------------      327
                            :* ********:  **:****:***:**  *

PVX_082400                  SNATNPSNAANCANSGNAAKIFGPPREYRFPATEKY---RDPELGAKAVRINMSGFQSHE      361
PBANKA_1344100              ---------------GNG-DNY-DDEYYYFPSCDKF---KQKE-NVKQIKIDLKRFKDHI      321
Py17XNL_001303339           ---------------ENDDNNY-DDEYYYFPSCDKF---KQKE-NVKQIKINLKKFKDHL      322
BOVATA_017340               ------------------------KYQFAQPDAS-----GDESGTPWEFDLSTF--RG      306
EfaB_PLUS_276.g39           -----------------------GGVYTPQIAVGDAVGEAAEQGESPISLDMSLYESRE      364
Cvel_22959                  -----------------------GGKRLFTTP-------------AGDTLDLTAYNSFD      308
```

Insert 3

```
TgMyoF                      -----------------------GGIYYFPSPKFR-----KAADAKAQEMDMSLFEPRD     356
Ncaninum_LIV_000262900      -----------------------GGIYYFPSPKFR-----KAADAKPQEMDMSLFEPRD     358
                                                                             :::. :

PVX_082400                  HFRYLTKSSVHELSDVNELEQFETTVYAMQTIGISEEEQHQIFRVLEGILYMGNVLFVND    421
PBANKA_1344100              NFRYLTKSSVYELNDVNELEEFEATVYAMQIVGIEENEQNQIFKILEGILYIGNILFNND    381
Py17XNL_001303339           NFRYLTKSSVYELNDVNELEEFESTVYAMQVVGIEENEQNQIFKILEGILYIGNILFNND    382
BOVATA_017340               NFRIVPPDSER----DFDLPTFDETLAALRTVGFTYEQVSVVFDIVATVLHLSNIEFVEK    362
EfaB_PLUS_276.g39           HFKFLTCSSCYQLVGIDDADAFNTTLKAMLTLGMSAETIQNIFQVVAAILCLGNTSFSVD    424
Cvel_22959                  VFKYLTKSSCSELDGVDDVEQFSRTVGAMSTVGISAEEQQSIFRVVAAVLFLGNVNFEAP    368
TgMyoF                      KFKYLTKSSCHQLQGVDDCEEFESTLFAMQTVGISPEEQMSILSVVGAVLCLGNVSFETP    416
Ncaninum_LIV_000262900      KFKYLTKSSCHALQGVDDCEEFDSTVFAMQTVGISPEEQMNIFSVVGAVLCLGNVSFETP    418
                             *: :   .*         :    *. *: *:  :*:  :     :: ::  :* :.*  *

PVX_082400                  E-SREECRILDDSLSDLKMAASFLDVDAEELRDALCYRTIVANNECYKKPVNAVVANDVR    480
PBANKA_1344100              D-NKEESCILESTYEDLNNAAYLLDIDVDTLKDALCYKTIIANNEHFKKPVTSAMASDIR    440
Py17XNL_001303339           D-NKEESSILESTYEDLKNAAYLLDIDADTLKDALCYKTIIANNEHYKKPVTSAMASDIR    441
BOVATA_017340               --GSEGAMISNMEAGHCQTVTTLLNVDNVSLMNALMTRTIKTANEMYTKPLRVEEACDVR    420
EfaB_PLUS_276.g39           ADDSEAADVASSSLVDLQKAAKMLGVPAESLKEAMCFRTIKTARESLRKPLRKEEATEMR    484
Cvel_22959                  PGNSEASEVVDSCAPFLKSAAQILEVDTQALAEVLTIRTIRTRGEVFKKALLVHEADETR    428
TgMyoF                      KANSEGSQVAASCTEYVCKACRLLGVQREALQEAMCYRTIKTAHESYRKPLKTDEAWEMK    476
Ncaninum_LIV_000262900      KGNSEGSQVAPSCAEYVSKACRLLGVESDALQEAMCYRTIKTMHESYRKPLKTDEAWEMK    478
                              . * . :          .  :* :    * :.:  :** :  *   * :    * : :

PVX_082400                  DALARAIYGCLFLFLKVVERTNESVGFLEDA------------------------------    509
PBANKA_1344100              DALARAIYGCLFLKLVERTNESIGLIKDV------------------------------    469
Py17XNL_001303339           DALARAIYGCLFLKLVERTNESIGLIKDV------------------------------    470
BOVATA_017340               DAIAKNVYSMLFDHLVERINESIGYVPDA------------------------------    449
EfaB_PLUS_276.g39           SALCRALYGCLFLRVVELTNNSIGFISAEAAGAEAAAEAAAATAAEAAKAVTAGEVARKG    544
Cvel_22959                  DALARALYGTLFLRVVQRTNQSIGYLKEV------------------------------    457
TgMyoF                      DALCRALYGCLFLQVVAKTNASIGYLKEV------------------------------    505
Ncaninum_LIV_000262900      DALCRALYGCLFLQVVARTNQSIGYLTEV------------------------------    507
                            .*:.: :*. ** ::*   * *:* :
                                                          [Eimeria insert]

PVX_082400                  -------------TLLFCGVLDIFGFESFAVNSFEQLCINYTNECLQQFFNNFIFKCEEK    556
PBANKA_1344100              -------------NLFCGVLDIFGFESFPVNSFEQLCINYTNECLQYFFNNFIFKCEEK    515
Py17XNL_001303339           -------------NLFCGVLDIFGFESFPVNSFEQLCINYTNECLQYFFNNFIFKCEEK    516
BOVATA_017340               -------------NLTTGILDIFGFECFKFNSFEQLCINFTNETLQNFFNNFVFRCEEE    495
EfaB_PLUS_276.g39           PSVSKKERQQQKQLLFCGVLDIFGFECFQQNSFEQLCINYTNERLQQFFNSFVFLCEEE    604
Cvel_22959                  --------K------LNCGVLDIFGFECFQYNSFEQLCINFTNERLQQFFNTFVFKCEEE    503
TgMyoF                      --------QSADDLLLFCGVLDIFGFECFQFNSFEQLCINFTNERLQNFFNTFVFKCEEE    557
Ncaninum_LIV_000262900      --------KSPDDVLLFCGVLDIFGFECFAFNSFEQLCINFTNERLQNFFNTFVFKCEEE    559
                                          *   *:*******.*  *********:*** ** ***.*:* ***:

PVX_082400                  LYVDEGIEWDPLDFPDNVDSVNLLESKPYGVFCMLDEECYVPSGKDRTFCSKIISKHCSP    616
PBANKA_1344100              LYIEEGIKWSSLDFPDNKDCVNILQSKPFGIFCMLDEESFIPGGKDKTFCNKIISKHAN-    574
Py17XNL_001303339           LYIEEGIKWSSLDFPDNKDCVDILQNKPFGIFCMLDEESFIPGGKDKTFCNKIISKHVN-    575
BOVATA_017340               LYSAEGISWNALDFPDNSDCVDLFKSRPYGLFAMIDEECNLPGGRDQSLCNKIVQRHS--    553
EfaB_PLUS_276.g39           LYRKEKIQWSPLDFPDNADCVALLQDKQTGVFAMLDEECVVPAGSNRGYTLKLVKKHSSS    664
Cvel_22959                  LYCKEKIPWDPLDFPDNQDCVDMLQDKPHGIFPMLDEECIVPQGSDRSFCSKLLKQHS--    561
TgMyoF                      LYRAEGIQWNPLDFPDNADCVALLQEKPLGLFSMLDEECMVPAGKDRGFNNKVCQKHG--    615
Ncaninum_LIV_000262900      LYRAEGIQWNPLDFPDNADCVALLQDKPLGLFSMLDEECMVPAGKDRGFNNKVCQKHT--    617
                            **   * * *. ****** *.* :::.:  *:* *:***. :* * ::    *: .:*

PVX_082400                  G---------------GSGTSRFMAVKTDPSSFVIIHFAGKVTYNSSGFLEKNKDQLSAD    661
PBANKA_1344100              -------------------NKRFESIKTDPNSFIIVHFAGKVMYNSCGFLEKNKDQLSDD    615
Py17XNL_001303339           -------------------NKRFESIKTDPNSFIIVHFAGKVMYNSCGFLEKNKDQLSDD    616
BOVATA_017340               ------------------NNARFAKVKLDQSSFVVNHFAGAVQYKIDGFMDKNKDQLSND    595
EfaB_PLUS_276.g39           SSGGSSSGGSSSGGSSSGGSTRFKVNKTKQNCFIVKHFAGEVEYCTDGFLEKNKDVLSLD    724
Cvel_22959                  ------------------AHKRFDPVKTKPTWFTVNHFAGPVKYCSDGFLDKNKDQLLQD    603
TgMyoF                      ------------------GHKRFGVIKTKPNCFVVHHFAGSVEYCSDGFLEKNKDQLSVD    657
Ncaninum_LIV_000262900      ------------------GHKRFGVIKTKPNCFVVHHFAGSVEYCSDGFLEKNKDQLSLD    659
                                                  **   *  . . * : **** * *   **::**** *  *

PVX_082400                  VQKVLLHSESEYTSSLFQKHLRRN----------------VEKKRIQTVSSEFKEQLHQL    705
PBANKA_1344100              AQNVLLQSQNEYIHNLFKKYLRRN----------------FEKKRFVTVSSEFKQQLDVL    659
Py17XNL_001303339           AQNVLLKSQNEYIHNLFKKYLRRN----------------FEKKRFATVSSEFKQQLDVL    660
BOVATA_017340               AVSFILSTKIDNMRSIFQSYIDKKATAVRPG------GRAGGVTKQKTICTQFSGQLDSL    649
EfaB_PLUS_276.g39           LQQCIAAAANPFVAALFLSFLHRGGLETDGDKQQQQQQDGKRKRKVVTVSGEFREQLQTL    784
                                                                                         [Loop 2]
```

Journal of Cell Science • Supplementary information

```
Cvel_22959                VQECVKKSSFTYVADLFSELLNRGGGAGAAPA-----DPRVTKKKSNTVSSEFKEQLSEL      658
TgMyoF                    LQEAVKASTIPFVSNLFSAFLNRGTAEDGS----------GKKRKFVTVSSEFREQLGAL      707
Ncaninum_LIV_000262900    LQEAIKASSIAFVSHLFTAFLNRGASEDGASV--------GKKRKFVTVSSEFREQLGSL      711
                            . :  :       :*   : :                 :  *:. :*  **  *

PVX_082400                MRRIKETEPHFIRCIKPNSQNVPDLFDRISVNEQLKYGGVLQAIKVSRSGYPVRLTHTDC      765
PBANKA_1344100            MTRINQTEPHFIRCIKPNSKNVPDIFDRHSVNEQLKYGGVLQAIKVSRAGYPVRLTHQNC      719
Py17XNL_001303339         MTRINQTEPHFIRCIKPNSKNVPDIFDRHSVNEQLKYGGVLQAIKVSRAGYPVRLTHQNC      720
BOVATA_017340             MTKIGATSPHFIRCIKPSPECRPNHFNRKVVDSQLRCSGMLQVVQVSRAGYPVRFPHAEL      709
EfaB_PLUS_276.g39         METIGETNPHFIRCIKPNPKNLPDFFDRPSVNEQLRYGGVLQAVQVSRAGYPVRLSHMEA      844
Cvel_22959                MTAVGATDPHFIRCIKPNPQNLPDEFDRKSVTEQLRYGGVLQAVQVSRAGYPVRVAHTEA      718
TgMyoF                    METVDKTAPHFIRCIKPNPQNLPDLFDRATVNEQLRYGGVLQAVQVSRAGYPVRLSHRDC      767
Ncaninum_LIV_000262900    MDTVNKTAPHFIRCIKPNPQNLPDLFDRVTVNEQLRYGGVLQAVQVSRAGYPVRLSHRDC      771
                          *  :  * *********. :  *: *:*  * .**: .*:**.::***:*****. * :

PVX_082400                VRDYSVLLPKEGRELFR-G--------YAAKSWAQRATFVLSQLHRCDAIQDLLRSLGAR      816
PBANKA_1344100            INEYYILLTKEEKTAFSKH--------YGDKSLSEKANYILSKLENRDKIQDYIKSLKHI      771
Py17XNL_001303339         INEYYILLTKDEKINFSKY--------YGDKSLSEKANYILSKLENREKIQDYMKSLKHI      772
BOVATA_017340             FNSFRYLLEAKEADSAMQIA-----------DKRKLGEFVLNIL-----V----------      743
EfaB_PLUS_276.g39         VKEYRPLAAQQLQRQLLQQQHQLQQQQQQQKGWKEMAAILLEELHVSLSLNA--------      896
Cvel_22959                FDDYCVLAEKPVRTGLRKV-----------AAPKDRAQKLLETLTLSLPIPKLED-----      762
TgMyoF                    FFDYRALADGALAAQL---S----QGTETPEAWRGRAEALLRHLDEKLKL----------      810
Ncaninum_LIV_000262900    FFDYKALADKAVLEKLCMQS----EGTVSSETWRERAQALLLHLDAKLNL----------      817
                          . .:  *                                  .  :*  *      :

PVX_082400                RRAEEAAANTASE---------ANTADTANE-GNLANAADLATPESALLWAVGKSLCFFK      866
PBANKA_1344100            RQTQNQENIFYGSVLKKQYSKNPNLIEKKNITQYENNLSNNVIDNDKFIWYVGKTLSFFK      831
Py17XNL_001303339         RQKKNQENIFYGSVLKKQYTKNSNLIEKKNNTRYENNTLNDIIEDDKFIWYVGKTLSFFK      832
BOVATA_017340             -------------------------------TRY-----MTSKPPEEGSLAVGKTLIFMK      767
EfaB_PLUS_276.g39         ----------------------------------------SHEGHTGATWAVGETLCFFK      916
Cvel_22959                ------------------------------DPRR-----LEGQRGKGNTWAVGETLCFFK      787
TgMyoF                    -------------------------------DRR-----KKETDSPDRTWAVGKTLCFFK      834
Ncaninum_LIV_000262900    -------------------------------DRK-----KKDAPSHDKTWAVGKSLCFFK      841
                                                                        **::* *:*

PVX_082400                SDAYSVLSALRSDLRAAQAVVIQKNYKAYRQRRLFLTMKRKVVLLQRWARHILVVLQNER      926
PBANKA_1344100            IEAYNILLTMRQDFRSLNAVIIQKNYKCYIEKKKYKLLKKKIVTIQRWIRNILIILKKKK      891
Py17XNL_001303339         IEAYNILLTMRQDFRSLNAIIIQKNYKCYIEKKKYKLLKKKIITIQRWIRNVLTILKKKK      892
BOVATA_017340             NGPYEQVCLAMQGLRNRSAIKIQARVRCNIQRKRYLEALWSIRTFQIWVRYKIKKLQRQR      827
EfaB_PLUS_276.g39         RECFETLSSALVCKKSAAATKIQASFKGYERRQAYLGLKRDTVVIQRAVRAWLARRERER      976
Cvel_22959                QEAFEVLNTKRLMLRNKCATKIQARWKAVVGRRQFLEMMRNVRKCQAVARGALARLRVRN      847
TgMyoF                    NEAYEILSANLMSVRVAAATAIEARYKCFVQRRFFLMYRQTVVFLQSHIRMFLCKLEAQR      894
Ncaninum_LIV_000262900    NEAYEVLSASLMSVRVQAATAIQARYKCFVQRRFFLMYRQTVVFLQSHVRMFLCKLEAWR      901
                             :. :       :   *   *:    :    :: :           *    *   :    .   .

PVX_082400                TKVQRATQLICLHIYGYTVRRSFLRQKKCATIIQAHVRGYLARRHFQHYRVNHYASLIKA      986
PBANKA_1344100            IIMINAQKLICSYIYAYALRKIFLYKRKCAIIIQSAFRGYLIRQSYKVYRKNYYASKIQA      951
Py17XNL_001303339         IIMINAQELICSYIYTYALRKKFLYKKKCAIIIQSAFRGYLIRQSYKIYRTNYYASKIQA      952
BOVATA_017340             AIRTQAIMLIQSMYRMYVERKLMRELREKVARLQSLWRSVNSRVQTEEKRIHTMATKLQA      887
EfaB_PLUS_276.g39         QRLNAMACKIQRFMKMVVQRNKYLRLLKSITRLQALWRGRQARAASRDLRAHRAASRIQA      1036
Cvel_22959                IRKRKAAVRLQSYARMRRDRKRFLRFRKAVCSLQAKWRWKQFRRAFAQIFQQRKAAAIQA      907
TgMyoF                    LRESRAARRVENFMRGAVARLRYLRTLENIRRIQAAWRGKQTRSQLRDRKLEEAASKIQA      954
Ncaninum_LIV_000262900    RRQDRAAKRIETFLRGAVARLRYLRTLKQIKTIQAAWRGKQTRSKLRDLQLHEAAGKIQA      961
                                   :         *        :    :*:   *     *         .   *   ::*

PVX_082400                TWKMHKQRRHMANMKRAAEIIQLKWKGILARRQLRRLKEEAKEVGALIKKNQCLVKQINQ      1046
PBANKA_1344100            IWKSKKERIRYLKLIESTKKIQLKWKGILARRQLRRLKDEAKEVGALLSKNQVLMNELKN      1011
Py17XNL_001303339         MWKCKKERIKYLKLIESTKKIQLKWKGILARRQLRRLKDEAKEVGALLSKNQILMDELKN      1012
BOVATA_017340             AWKGYKCRCYYLELRSATIKAQLRWRSICARRTLRSLRMEAKDLGNVIKRAQVLEEDLKK      947
EfaB_PLUS_276.g39         TWKMKKDRAEFIALKQGTIKTQLRWKRMLAIRQLRQLKSEARDVAGLVKLVQGLKQELAE      1096
Cvel_22959                LYRGRKQKVYFEKLKKAVRMAQNKWRSILARRQLRRLRQEQKEAGALLSKVQELQTELQK      967
TgMyoF                    FWKMHKQRMFYTNLKKASTIAQLKWKRILARRMLRRLREEAREVSGLLKKAQDLQRDLCE      1014
Ncaninum_LIV_000262900    TWKMHRQRASYRDLRKAATLAQLKWKRILARRMLRRLREEAREVSGLLKKAQDLQRDLGE      1021
                           ::   : :      :   .     *  :*: :  * *  ** *: *  :: .  ::.   * *   :: :

PVX_082400                ERKKKMEAEHKLLQAFASVEKLAKRVDLLERNNRENENVIKGLMERLAQANAPPS-----      1101
PBANKA_1344100            EKSEKIEIENKLLKASANAQKLTKRIEVLEQINKNNEVLIKNLIEKVENLSLKQK-----      1066
Py17XNL_001303339         EKSEKFEIENKLLKASANSQKLTKRIEALEQINKNNELVIKNLVEKVEKLSLKQK-----      1067
BOVATA_017340             EKAMRADAEARVLQLTAKLSTIEKSLEELRGQVDTLTKERDGLAGRLHEAETNTQ-----      1002
```

Plasmodium Insert

Journal of Cell Science • Supplementary information

```
EfaB_PLUS_276.g39           ARQQIEDAATRELQQQARADELQKQLQCTKKEALELKTELRGNAEALAAQHRQLE-GLR-      1154
Cvel_22959                  HKKEASEAEARSFYVQAEVDKKDSLIASLRKEIEQLKKSLEDAETQRVEMESHLEEALKQ      1027
TgMyoF                      EKNKRSDAESHVLQLQAKNEDLLKEIQKLQRELERAKEDVASLQASNDDFASQ-------      1067
Ncaninum_LIV_000262900      ERSKRSEVESHVLQLQAKNEELLKEIQRLHKELDRAKEEVASLQASNEDFASQ-------      1074
                             :    :   : :   *  .   . :   .           .

PVX_082400                  --SETPISEIPSERTRIRAAPPPRRDDPAVEASTAATVATAATAATATSATTSAAAASPE      1159
PBANKA_1344100              --EENTEK----------------------------N------ISNEHKKYISDNNEISR      1090
Py17XNL_001303339           --GEIAEKGAG----------KNAEENAGK------N------AEENAGKNISDNNKISR      1103
BOVATA_017340               ------------------------------------------------------------      1002
EfaB_PLUS_276.g39           --QLLNRSDSGLAFAD-EAVPTPSEGM---------QL-----TPAA-------------      1184
Cvel_22959                  QTKTEKEKEEAVKAAAVVAQAAPA-AA---------T-------P--TSREVEAHVMEAL      1068
TgMyoF                      -VKQLKES---L----TVGSSTPT-TP---------QM-----TFGTHKRRVSNNADVPH      1104
Ncaninum_LIV_000262900      -VKQLKES---L----TAGSSTPS-TP---------QM-----TPGTQKRRLSNHADAQQ      1111

PVX_082400                  RGTPAQGE----QMESLLSKIKQLELENKEHLKKNNALNERYQRLLGLLSH-FR------      1208
PBANKA_1344100              KQDSMSDQ----NLSKLLDKIKKLEIQNEEYIKKNTLLNERYNKMLNIFSY-FK------      1139
Py17XNL_001303339           KQDSGSDH----NLFKLLDKIKKLEIQNEEYVKKNALLNERYNKLLNIFSY-FK------      1152
BOVATA_017340               --------KAQTDLRMIKEFV---SKEAVSG--NQSDW---LSNVLGGQ------GVER      1039
EfaB_PLUS_276.g39           --ARGTRHSAGAEAEELRELFAKRQKEAAAKEHEQTVLLQQLQKQVQELQQQLQQEQQQR      1242
Cvel_22959                  QQAREEGQRAETRANTLAQEKENIEKDFNRLAEEHEALKGQYQTLLEGARLQ------QR      1122
TgMyoF                      SQEK-DKFPTDEELKALRTELERRDAEAQQQQAEHEALIAELRAALKDAESACEYERTQR      1163
Ncaninum_LIV_000262900      SQGDRLSTQTDEELKALRQELEKREAEAQLQQSEHETLIAKLQASLKEAESALEQEKTQR      1171
                                            :       . :      ::          :

PVX_082400                  -----RKDVCLEGGEAARELL--SGH------AAKVLQR---GSLSEWRPVEKRAN-RSG      1251
PBANKA_1344100              -----GKHNLINEANEKNIPNNVKKD------QLNMINSILYNEMYEQNFIKNQSHINQT      1188
Py17XNL_001303339           -----GKHNLINDSNEKNIPNNMKRE------QLNIINSMLYNEIYEQNLMGNKIDINYG      1201
BOVATA_017340               A---------TGDTSAAAA----------ASRTG---------SM---------------      1056
EfaB_PLUS_276.g39           QQAEAKYKLVLEDSSVRRDREEPREP-----QQDKA--------V-----VA--A-----      1277
Cvel_22959                  QQG--SIDL-RRDSSAASVEKDQPPTTPAAAKPEK---PSEPTPAPTPKVAA--ADTPKP      1174
TgMyoF                      KEAEARYRLVLEDSSASSTHVRASSASKAAVADS----------APTA--LL--REKCME      1209
Ncaninum_LIV_000262900      SEAEARYKLVLEDSSASSTHFRLSSAGAAKQQPGSSLASGEAGGVSST--LL--REKRNK      1227
                                          .
```

→ Region of unknown structure

```
PVX_082400                  DDGDDGDHHDHYD-H--RDEHTAPP----NARRTVHRPNGRDVDILMCGPKGVGKTSLLE      1304
PBANKA_1344100              P-SVVKDKKEIF--DNNLTEFKEHQ----NLTKVGNKTRDNVIDILICGPKGVGKTSLVE      1241
Py17XNL_001303339           SSSIVKDKKEMSD-NNNITEFKEHK----NLTKIGNKTRDDVIDILICGPKGVGKTSLME      1256
BOVATA_017340               ---KLERHS-------SRSLHSPPGV--------RTKSIEHGADIVLCGPPGCGKTRLLE      1098
EfaB_PLUS_276.g39           --QVTRKQASGLEES------------AIRHAETTTVCDRRFIDLMLLGPPMVGKRDLLK      1323
Cvel_22959                  SDAPVDRPADGAET-------------TRQRRSAPHVHDGKPYDIVVLGIEGSGKTSMLE      1221
TgMyoF                      SDMATGRPGDSLASSVASAAPLSPPVALEPELWESVLHDQRWIDLLLLGPAGVGKTGLLE      1269
Ncaninum_LIV_000262900      V-----PDGDGAL-AATKTLSPLPSVALASEHWECVLHDQRWIDLLLLGPAGVGKTGLLE      1281
                                                                       *::: *    **  :::

PVX_082400                  DLFVRIGDEINLNLLRKNKK-KNANESNSFV--YD-TYVVAH-----------KSSSVKI      1349
PBANKA_1344100              DLFVRLGDENNLNILRKKNKKKEIDGINHPN--YN-TYIVNH-----------KLSQIKI      1287
Py17XNL_001303339           DLFVRLGDENNLNILRKKNKKKEIGNINHPN--YN-TYIVNH-----------KLSQIKI      1302
BOVATA_017340               MALIKKGDDKNLQLLRNVDEARSR-KRRMAPTAFEFTVSPGH----------------SI      1141
EfaB_PLUS_276.g39           ALVEQLGDTSALMQLRGPA--DDDSTFEQLV---KVKLPDG-------------SRLVC      1364
Cvel_22959                  QFCTSMGNEEGPKKLRRAK--EEDAPHVLIPVPFQGK--------------------EL      1258
TgMyoF                      QFLVKLGDEVHLEQLRVSRKMEDQAPFSKLPQHYELVYSPDSQEGH-------EREEARV      1322
Ncaninum_LIV_000262900      QFLIKLGDEVHLEQLRVSRKMEDQAPFSKLPQHYELVYPRDSEDAPTRGAKPRDDELTRV      1341
                                  *:      **      .           .

PVX_082400                  CDYMYSGSPSAEEGLFNLVKSSASIVVVFDSSNGDSIHPALHLLQEAALTNV--------      1401
PBANKA_1344100              VDCGYSDNTNSEEILFEYIKNSICIIIVFDSTNKESIIPALHLLQEASLINV--------      1339
Py17XNL_001303339           VDCGYSDNTSSEEILFEYIKNSICIIIVFDSTNKESIIPALHLLQEASLINV--------      1354
BOVATA_017340               SVTEIPGSYFDSDDARTILRNAHLVAICFDPGNQQTYDDARVIVKL-----------LKQ      1190
EfaB_PLUS_276.g39           LCFTFDETKHNLEAVKQRACQAFVIACIFDPTKRATYGTCLSLLQDAVIPAIKEAG----      1420
Cvel_22959                  RVLKCSGKKQYYAFVRDQLARAKWVFAVYQPSMDNQ--EIFAFLEKVL--------R-DA      1307
TgMyoF                      NVLDFPGLSRTKQNPALRVKQAFVVAVVFDPTRPETCAEALQILTNVVLPARPKVSAQDP      1382
Ncaninum_LIV_000262900      NVLDFPGPSRTKQNPALRVKQAFVVAVVYDPTRPETCDEALQVLTNVVLPCRPKVAAQDG      1401
                                                 :  :   ::            .:

PVX_082400                  ---KRRTKLYLLENIFNEKINMKKNACDVSYSLRV---AKACNAHYVKALDMYEIVNNYV      1455
PBANKA_1344100              ---KKSTKLYLLENIFNEKINLNPNVNDVSYAQKV---SKTCNATYIRALDIYDILNDHV      1393
Py17XNL_001303339           ---KKSTKLYLLENIFNEKINLNPNVNDVSYAQKV---SKTCNATYIRALDIYDILNDYV      1408
```

```
BOVATA_017340               LVDCKNTRICLVQNDYIILESARPIVCDINQVQTFA---VENDLLYMRIRDLMEFVEQ--    1245
EfaB_PLUS_276.g39           -GGLPATKVFLVENNSNTT-SKGAIEVDVEKARDE---VASLGCHYKALMQLSSLYDDLK    1475
Cvel_22959                  --MAAGCRILVIGNTWHVQRGQ-EWKVDLISVKDL---AARHRCYAVETA---SFSDA--    1356
TgMyoF                      LGACISGRVYLVENGWRAAAKEAAVQVDTAAVRDK---AAGLRCHYRELVHLESLVDEIV    1439
Ncaninum_LIV_000262900      LAACVSGRVYLVENGWRVAAREGTVQVDTAAIRDKGTSTAGLRCHYRELVHLESLVDEIV    1461
                                   :: :: *             *                         .: :

PVX_082400                  CGARSYL----GSFPGQTYPMQ-----------------------RGGDLSPWRHCER-    1486
PBANKA_1344100              NRMTTSSTYFLNNFISEESISKDMFISSKHFNTY-----DNNIIEYSETANSRWFQNENN    1448
Py17XNL_001303339           NRITTNSTYFLNNFISEETISKNMFINSKHFHTHDSSNNDNNIIEHLDRSNSRWFQNENR    1468
BOVATA_017340               -----VTGYV-ESKRGQ-------------------------------------------    1256
EfaB_PLUS_276.g39           PWLSALAEYR-RSKPEQ----------TKHRRISK-------------------------    1499
Cvel_22959                  -LFMVWETFT-AP--------------------VPQ---------PAQPAVQPIRHPARN    1385
TgMyoF                      PLMSSWRDLL-QQQRLAM------L--QHHARLQPG---------GPSPHASPFSLPRSQ    1481
Ncaninum_LIV_000262900      PLMAEWRGML-QQQRLAM------L--QHHARLQPG---------SAA-GLSPFALPRPP    1502


PVX_082400                  YHHVARQIGEGP---SPGDYISAYAHAYEYADVDAHSHARALKMGDPGKSAGETPAHVNH    1543
PBANKA_1344100              YKQY-IKNGNNLIFDKTDGYFQHFNKSD--------------------KFQ---EDKINL    1484
Py17XNL_001303339           YKEN-IKNGNNCILGKTDEYFHNFDKFD--------------KFGKFDKIQ---EGEINL    1510
BOVATA_017340               ---------------------------------------RAAGAGD--------------    1263
EfaB_PLUS_276.g39           -------------------QHYA------YTPS---QQPHRTGTGF--------------    1517
Cvel_22959                  VA-------PQEHRPRPTDTPAA------GEPS---G-----------------------    1406
TgMyoF                      -------------TGRGASLFAT------ASQS---GALGASAVGGQENGVE--------    1511
Ncaninum_LIV_000262900      AQ-----------ASRGASLSFS------NA-------AHAAGSGLRENGAEKSP-----    1533


PVX_082400                  LNLPPEGECPPNHFSPKHFPPKRDQQTAHP-NQQNSVSSMVSSFKS-I------------    1589
PBANKA_1344100              LHHQSSTHS------VRNMQNTKDMENKNLIYLKNESDIYRESYDKFS------------    1526
Py17XNL_001303339           LRHQSSTQS------VNNIQDNKDIENKNLIYIKNENDIYRESYDKYS------------    1552
BOVATA_017340               --ASRS-GRTPN--------------RGALS-------LFYEKFRNFWAGVNYS------    1293
EfaB_PLUS_276.g39           -----------M--------------RGSGSFSA----TLVDSIRSFISSGKSSYEA---    1545
Cvel_22959                  -------EAEER--------------KGGIMNIG----AMAQTLQTWFGKSPAA-GQGRE    1440
TgMyoF                      --KSSSGVSAHR--------------QGGLSA------NFFDSIRSFLSQSSMRPAGN--    1547
Ncaninum_LIV_000262900      -SVSGSASGTHR--------------QGSLSA------NLFDSIRSFLSQSSMRPGPGSA    1572
                                                                     ..  .

PVX_082400                  CGVTHKKNPHAQLLKESLPHNSYIY---GSKKYNVEMGKGLQPIFEVTLKGSVPITYLLI    1646
PBANKA_1344100              TKNVGKKKQVIQLLRESLPHNNYVY---NSKKYNPELGKGLQPVYEIMIKGNIPITYLCI    1583
Py17XNL_001303339           TNNLGKKKQIIQLLRESLPHNNYVY---NSKKYNTELGKGLQPVYEIMIKDNTPITYLCI    1609
BOVATA_017340               -------GFQNKLLVPTLVAQEGYK-----LPPL-----SLKRVANVN-NYTAAVTCLAF    1335
EfaB_PLUS_276.g39           TKKSMKLNQDLQFLLPSVPAK------DGQMPELSGPCDGVVPVIELTHETQGAITCLAF    1599
Cvel_22959                  REREREGLSQNVLLRPSLKGTESMRTQAGGKPRKEADWQDLRCVQEVT-ESESAVTCLCF    1499
TgMyoF                      VLASGKQAADLKLLRPSMKPGGTAMMK--KLKTRSLDQHSVVPVQELQ-DSDSAITCVVF    1604
Ncaninum_LIV_000262900      LASGAKQSVDSKLLRPSMKPGGSAMLK--KLKSRNMDAHSVVPVQELQ-DSDSAITCVVF    1629
                                        :*  ::                      .:  :  ::  .   :* : :
             Region of unknown structure <------  ------> WD40 domain
PVX_082400                  GQDSINKMHTLLAVGCKDGVIYIYKCSRTPLESAHGISGVTAVSS---------------    1691
PBANKA_1344100              GQNSINKNYTILAVGCKDGIIYIYKCFRTKMEQSDSFQTRGQGNGIVNNSSHEEEVIRNR    1643
Py17XNL_001303339           GQNSINKNYTILAVGCKDGIIYIYKCFRTKMEQSDTFQTRGQENETINNSNHEKELVRNR    1669
BOVATA_017340               RPEDPSDAYIVLAVGRRDGSINLYHCFRTETELRALNSGNFDESAPH-------------    1382
EfaB_PLUS_276.g39           GAEREHRDYILLAAATKAGQVMVYRCYRTEMEKNTLDPQELRRPTTQEE-----------    1648
Cvel_22959                  GQERHHLHYYLMACASKDGNIVIYRIYRTEMERAQMSKDDLDQIQSVSS-----------    1548
TgMyoF                      GKEKENRDYILLAAASKDGSVVIYRCYRLEAERQMFDQEQVSLVT-PAA-----------    1652
Ncaninum_LIV_000262900      GKEKDNRDYILLAAASKDGSVVIYRCYRLEAERQMFDQEQVSLLVAPAS-----------    1678
                              :     : ::* . : * : :*:  *   *

PVX_082400                  --------------VSSV-----------------------DGAGGEEGAPAKLVTKLSG    1714
PBANKA_1344100              KRGNLSNEEINNSELSTPEEE-QDENNTIEYINETIKDESSFALSEDNSMSTVLLSKLSG    1702
Py17XNL_001303339           KKGNLSNEEVNNSELSNPEEEEEDENNTIEYINETIKDESSFTLSEDNSMSTVLLSKLLG    1729
BOVATA_017340               --------------------------------------------PVDDNVLITESFTLAI    1398
EfaB_PLUS_276.g39           --LKINTEAMPNI-----------------------FVGKRASPLAETTDRIMLHSQMIG    1683
Cvel_22959                  --GQ---------------------------------SSKWDTAPPSEHSLVAIHSRLIG    1573
TgMyoF                      --S------------------------------------RTTDSKEHIGPSVSVHSRLVG    1674
Ncaninum_LIV_000262900      --K------------------------------------PPGDNREHFGPSVSVHSRLVG    1700
                                                                                     :

PVX_082400                  HKKAITCLVFSFTE-EKIISSSIDRTIKIWEVATGFLLKVFSDSSATLSVLLLPTNLDLF    1773
PBANKA_1344100              HRKAITCLVFSFSE-DKIISSSIDRTIKIWEVSTGFLLKVFSDSSATLSVLLFPTNLDIF    1761
```

Journal of Cell Science • Supplementary information

```
Py17XNL_001303339          HRKAITCLVFSFSE-DKIISSSIDRTIKIWEVSTGFLLKVFSDSSATLSVLLFPTNLDIF     1788
BOVATA_017340              HDKAVTCMCFSKVEVNELVTTSVDCTIRAWNVMSGQLIKVFNDSDPGLAVMFHPVDPTLF     1458
EfaB_PLUS_276.g39          HARAITSMFFTLLE-DHLVTTSIDCSIRFWVVNSGVNLKVFQDSAVPLAAALLPFNPSVF     1742
Cvel_22959                 HSRAVTSIFFSLLE-DQLVTTSIDKSVRFWSVDSGEMQKVFTDSSPALVAAFLPFNPKVF     1632
TgMyoF                     HSRAVTCLFFSLLE-DQLITTSIDKSVRFWHVDTGDMLKVFTDSSPALAAAFLPFNPTAF     1733
Ncaninum_LIV_000262900     HSRAVTCLFFSLLE-DQLVTTSIDKSVRFWHVDTGDMLKVFTDSSPALAAAFLPFNPTAF     1759
                           * :*:*.: *:  * :.::::*:* ::: * * :*   *** **   * . : * :   *

PVX_082400                 LCSNCTSLLRIVNVNTGHVNQKIKFESEIRTLEIDDTGLNIFAGSKNGTLYILEIVLNER     1833
PBANKA_1344100             LCSNCTSLLRIVNLNSGQVYQKIKVESEIRALEMDYTCLNIFAGSKNGTIYLLECLYNER     1821
Py17XNL_001303339          LCSNCTSLLRMVNLNSGQVYQKIKVESEIRALEMDYTCLNIFAGSKNGTIYLLECLYNER     1848
BOVATA_017340              ICCNANPTMRIIHYNQGTVLQKIRTKSELRCLVFDDTRFNCIAGNERGAICIYEAQADLH     1518
EfaB_PLUS_276.g39          IVSNNKSVLRLVCSNSGKVLQKMKMESEVRAIRFDDTGLFCFAGNKAGQLYVLEASDNAT     1802
Cvel_22959                 VAANSNAVLRLVNVENGIVHQKLKVDAEVRALKFDDTGLFCMAGTKTGNIHVLEASDNAN     1692
TgMyoF                     VASNSNSILRLVCATSGRVIQKLKVESEVRALKFDDTGLFCFAGTKAGAVHVLEASDTVN     1793
Ncaninum_LIV_000262900     VASNSNSILRLVCATSGRVIQKLKVESEVRALKFDDTGLFCFAGTKAGAVHVLEASDTIN     1819
                           : .* .  :*::    * * **:: .:*:* : :* * :  :**.: * : : *

PVX_082400                 VEIRFKLLFSLSPITCIRFVPKQPLLASPLIVVNSCDNHMGIIECVYGSKGAVLTSLSVK     1893
PBANKA_1344100             VEIKFRFLFSLLPITCIKFVPRKYIHTPPTIIVNSCDNHIGIIECMYGNKGI-ITNLSVK     1880
Py17XNL_001303339          VEIKFRFLFSLLPITCIKFVPRKYIHTPPTIIVNSCDNHIGIIECVYGNKGI-ITNLSVK     1907
BOVATA_017340              LKLSTTKSISRGPVTCVNFVPSSSPDVPPCIIANVCSGQITILNCVYDGSSGKIAEITYR     1578
EfaB_PLUS_276.g39          LTYKYKLDISRGAITCITFVPSRSPREWPLVLANCCDSNVAVVECVYGIPNGSLSNLQVC     1862
Cvel_22959                 LKFKFRLTVARGAITCITFVPSQDPGRNPMLLVNSCDSIVSIVDCMYGPAPGVLSSLQVQ     1752
TgMyoF                     IRFKFKTSLGKGAVTCITFVPSTGPGQYPRLLINCCDSSVAVVECIYGPPPGVLTNLLVR     1853
Ncaninum_LIV_000262900     IRFKFKTTLGKGAVTCITFVPSTGPGQYPRLLINCCDSSVAIVECIYGPPPGVLTNLLVR     1879
                           :       ..   :**: ***       * :: * *.. : :::*:*.     ::.:

PVX_082400                 HRIRINHALLPIRSSFSRFGGGWVVSGSEDGNIYVCSLLPHSNYRL-VFLKHHKAPVMAV     1952
PBANKA_1344100             HRIRINHALLPIRSCYTKFGGGWLVSGSEDGNIYVCSLLPQSNYKL-ILLKHHKAPVMSV     1939
Py17XNL_001303339          HRIRINHALLPIRSCYTKFGGGWLVSGSEDGNIYVCSLLPQSNYKL-ILLKHHKVSLEMR     1966
BOVATA_017340              YTVNNAHVALPVRSCYSRFGGGWCISGSEDRNLLIFSLLEENM---PYTISFHQGPVVAV     1635
EfaB_PLUS_276.g39          KRVKMKHEVLPLKNCFLHTGGGWLVSGCEEMGVFCYPVRERASAKNGFHLSGHAQAVLAV     1922
Cvel_22959                 HRVKVAHSLLPLRCCYSGFGGGYLISASEDKEVYVYALRKEMHYKS-AYLGHHRAPVLAV     1811
TgMyoF                     HRVRIAHSLLPLRCWFSNFGGGWLITGSEDKDVYCFSLQQGANFKA-ISLKHHQAPILAV     1912
Ncaninum_LIV_000262900     HRVRIAHSLLPLRCWFSNFGGGWLITGSEDKDVYCFSLQQGANFKA-ISLKHHQAPILAV     1938
                             :.  *  **::  :   ***: ::..*:  :    :           :  *   :

PVX_082400                 VVND--IDTLMVSG--------------DSKGNVVFWRRAFV---------------     1978
PBANKA_1344100             VVND--IDTLMISG--------------DSKGNIVFWRRSLI---------------     1965
Py17XNL_001303339          RPLYRTFICFILLYFALFFYFFYFYFFFFRPQSCQLW--------------------     2003
BOVATA_017340              AVNR--LDTLLVTS--------------DSKGSVAFWRRILVSSKA-----------     1665
EfaB_PLUS_276.g39          AVNA--QGTLLASA--------------DSCGSIILWRRLAAPKAS-----------     1952
Cvel_22959                 ATNV--SDSLLVSA--------------DSMGSIAMWRRFDFSHLL-----------     1841
TgMyoF                     ATNL--QDTLLVSA--------------DSMGKLVLWRSLDFSAAGGGAGGARGRAV     1953
Ncaninum_LIV_000262900     ATNL--QDTLLVSA--------------DSMGKLVLWRSLDFSGAADASAGARGRAV     1979
                                    ::                     .  :*
```

WD40 domain ←

Journal of Cell Science • Supplementary information

Fig. S1. Multisequence of alignment of MyoF amino sequence from Alveolates.

*Tg*MyoF (TgME49_278870) was aligned with sequences from *Plasmodium vivax* (PVX_082400), *Plasmodium berghei* (PBANKA_1344100), *Plasmodium yoelli* (Py17XNL_001303339), *Babesia ovis* (BOVATA_017340), *Eimeria falciformis* (EfaB_PLUS_276.g39), *Neospora caninum* (Ncaninum_LIV_000262900), and the unicellular algae *Vitrella brassicaformis (*Cvel_22959). Several differences between these sequences are notable. Insert 1 (orange box) is only found in *Toxoplasma*, *Neospora* and *Eimeria* species, while inserts 2 and 3 (orange box) are found in all species, although the size and sequence of the inserts vary significantly. For example, insert 3 from *Toxoplasma* is 22 amino acids in length, 46 amino acids in *Eimeria* MyoF and 68 amino acids in *Plasmodium vivax*, with no sequence similarity between these sequences. *Eimeria* and *Plasmodium* species each contain inserts not found in the other species (magenta and yellow boxes, respectively). The *Plasmodium* insert is 46 amino acids and is located near the junction between the motor domain and lever arm. Loop 2, a critical loop that interacts with actin, varies in both length and sequence (green box). *Plasmodium* MyoF has the shortest loop 2 at 16 amino acids. Loop 2 from *Tg*MyoF is 22 amino acids. Loop 2 in *Ef*MyoF is 32 amino acids and contains a glutamine rich insert. Each motor domains 6 predicted IQ motifs (highlighted using alternating green and orange text), although the primary amino acid sequence is poorly conserved. The unstructured mid region between the coiled-coil and WD40 domains vary in length between 413 (*Neospora*) and 214 (*Babesia*) amino acids (indicated with pink arrows and text). MyoF from each species contains a predicted WD40 domain (indicated with red arrows), the defining feature of this class of myosin motor. Residues that are predicted by AlphaFold (**Fig. S7**) to mediate WD40 domain oligomerization are highlighted in red.

Journal of Cell Science • Supplementary information

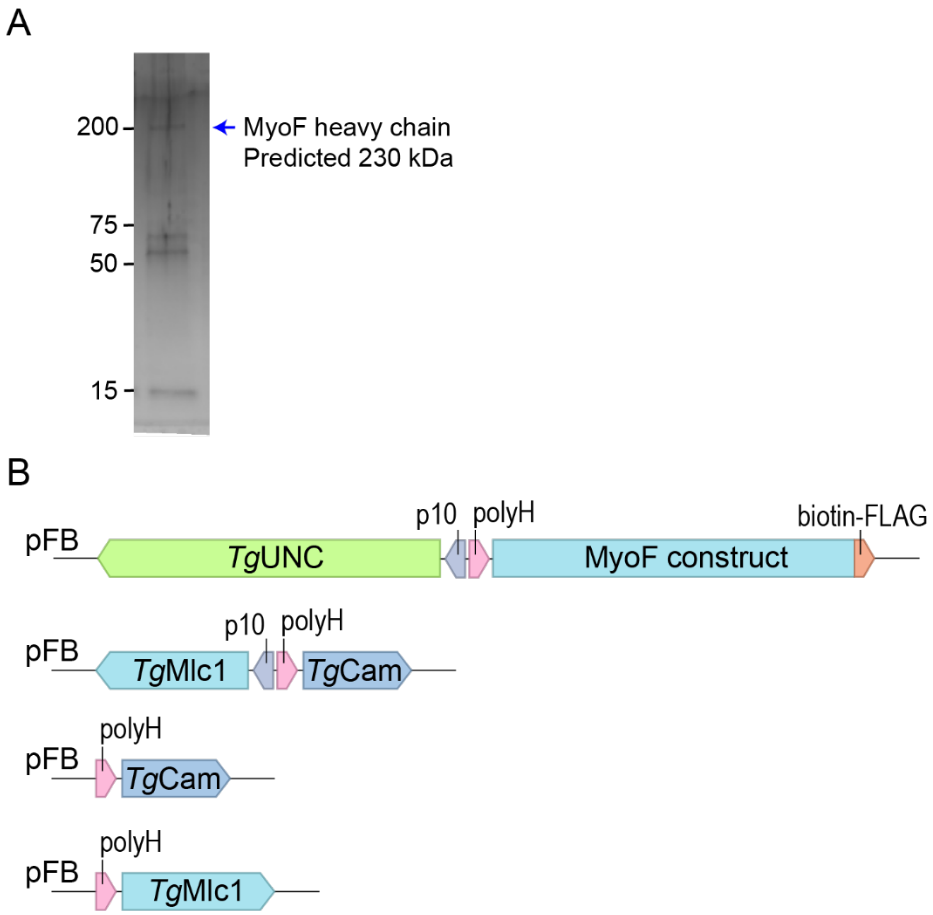

Fig. S2. MyoF immunoprecipitation from *T. gondii* cell extracts and strategy for expressing MyoF constructs in the *Sf9*/baculovirus system.

(**A**) Representative silver stained SDS-PAGE gel showing the elution following a MyoF-GFP immunoprecipitation assay from *T. gondii* cell lysates using GFP trap affinity resin. (**B**) Schematic of *Sf9* baculovirus constructs for the expression and purification of MyoF constructs, containing a C-terminal biotin-FLAG tag. MyoF constructs were expressed back-to-back with the *T. gondii* chaperone, *Tg*UNC from polyhedrin and p10 promoters. MyoF constructs were co-infected with *Tg*Cam and *Tg*Mlc1 expressed back-to-back or separately depending on the experiment.

Journal of Cell Science • Supplementary information

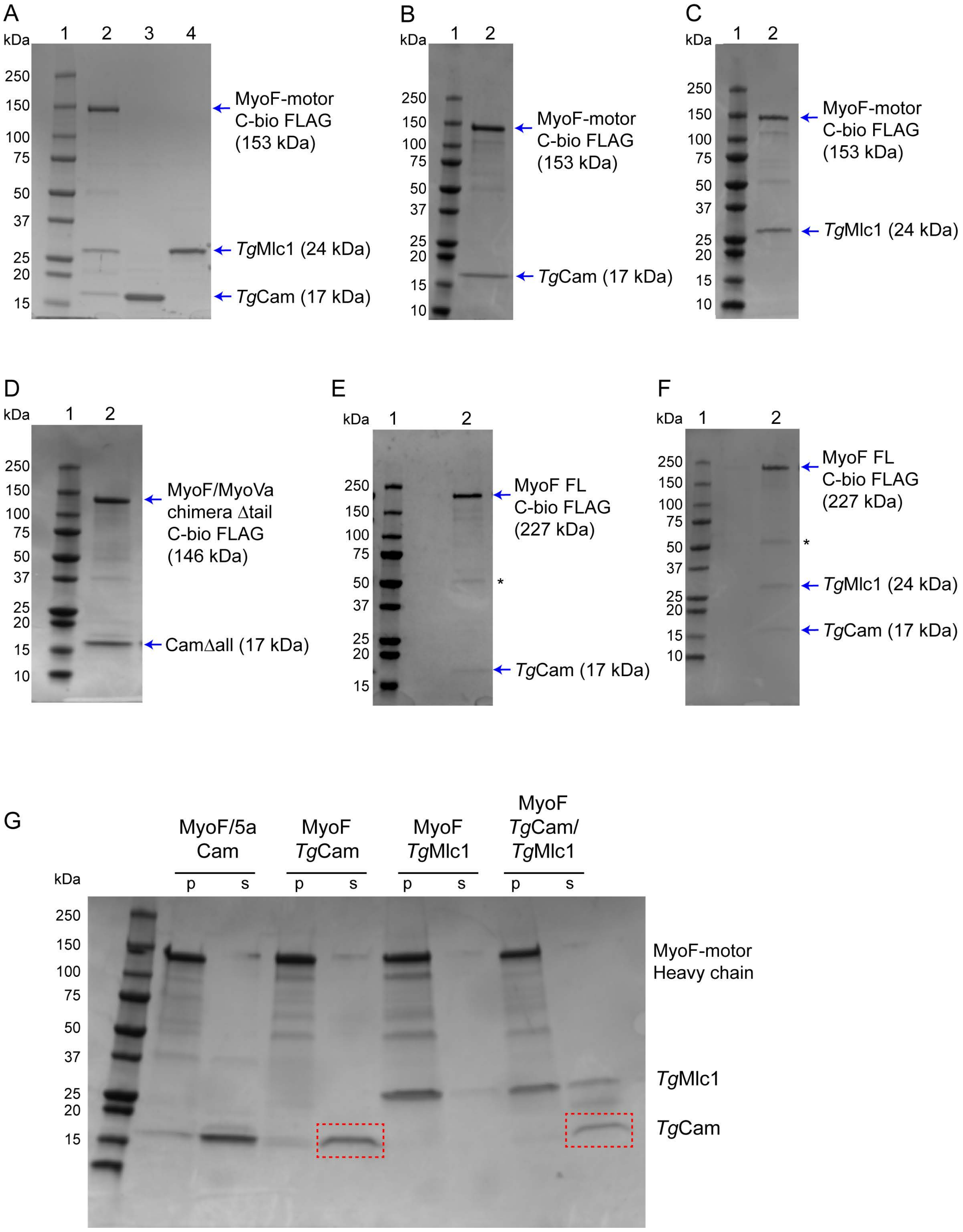

Journal of Cell Science • Supplementary information

Fig. S3. Coomassie stained SDS-PAGE gels of proteins used in this study and thermal denaturation assay to determine *Tg*Cam and *Tg*Mlc1 stoichiometry.

(**A**) Protein molecular weight marker (lane 1), MyoF-motor co-purified with *Tg*Cam and *Tg*Mlc1 from *Sf9* cells (lane 2), HIS purified bacterial expressed *Tg*Cam1 (lane 3) and *Tg*Mlc1 (lane 4). Data are from Fig. 1G. (**B**) Protein molecular weight marker (lane 1), MyoF-motor co-purified with *Tg*Cam (lane 2). (**C**) Protein molecular weight marker (lane 1), MyoF-motor co-purified with *Tg*Mlc1 (lane 2). (**D**) MyoF/Va chimeric construct co-purified with mammalian CamΔall (lane 2). (**E**) Protein molecular weight marker (lane 1), full-length MyoF co-purified with *Tg*Cam (lane 2). (**F**) Protein molecular weight marker (lane 1), full-length MyoF co-purified with *Tg*Cam and *Tg*Mlc1. Asterisks indicate contaminating *Sf9* tubulin identified using liquid chromatography-mass spectrometry (LC-MS). (**G**) Coomassie-stained SDS-PAGE gel showing (lane 1) molecular weight marker and supernatant (s) and pellet (p) fractions of thermally denatured and centrifuged (lane 2 and 3) MyoF/5a bound to calmodulin, (lane 4 and 5) MyoF-Motor bound to *Tg*Cam, (lane 6 and 7) MyoF-Motor bound to *Tg*Mlc1, and (lane 8 and 9) MyoF-motor bound to *Tg*Cam and *Tg*Mlc1.

Journal of Cell Science • Supplementary information

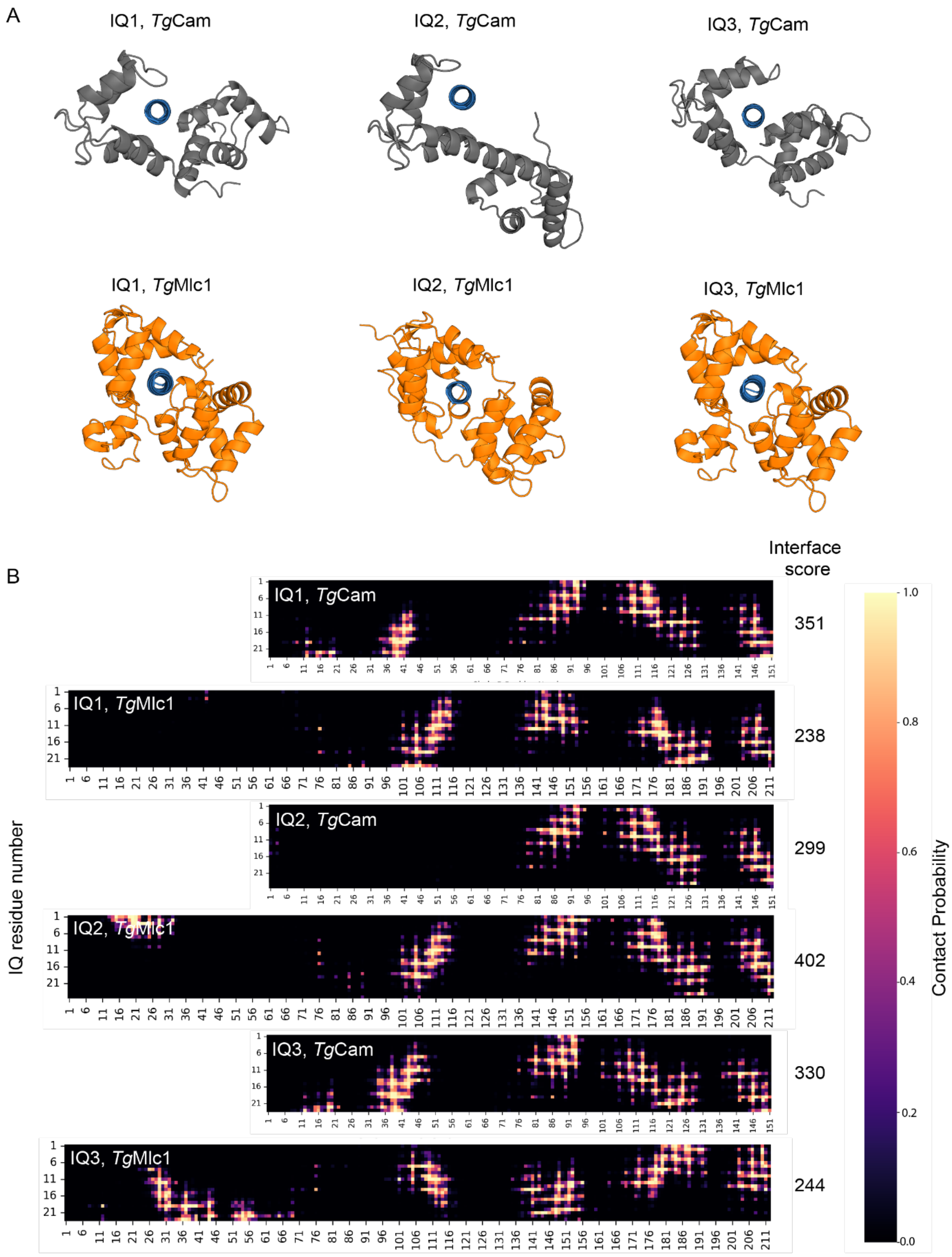

Journal of Cell Science • Supplementary information

Fig. S4. Computational prediction of *Tg*Cam and *Tg*Mlc1 binding to IQ motifs in MyoF.
(**A**) AlphaFold model for the binding of *Tg*Cam (grey) or *Tg*Mlc1 (orange) to the indicated MyoF IQ region (blue). Amino acid sequences for the first three predicted IQ motifs in MyoF are (IQ1: VRVAAATAIEARYKCFVQRRFFL; IQ2: MYRQTVVFLQSHIRMFLCKLEAQRL; IQ3: RESRAARRVENFMRGAVARLRYL). (**B**) Contact probability heatmaps and interface scores for *Tg*Cam and *Tg*Mlc1 binding to MyoF IQ motifs. For each IQ motif the contact probability was visualized using probability heatmaps which is based on minimum residue–residue distances. The distance between two residues was converted into contact probability using the following logistic function:

$$P(d) = \frac{1}{1 + e^{k(d-d_0)}}$$

where d = minimum distance (Å), d_0 = contact midpoint (e.g. 8 Å), k = steepness (e.g. 1.0). In this way P = 1 predicts a strong contact where P = 0 indicates no contact. Plots show the residue–residue distance matrix with amino acids numbers for light chains on the x-axis and IQs on the y-axis. Thus, areas of lighter shading indicate a high probability of interaction. From this analysis, a numerical summary was also generated (interface score) which is a sum of probabilities which is an overall binding proxy, where the higher the interface score, the greater likelihood of binding. Results of this analysis indicate that *Tg*Cam is predicted to bind more tightly to IQs 1 and 3 while *Tg*Mlc1 binds more tightly to IQ2 compared to *Tg*Cam.

Journal of Cell Science • Supplementary information

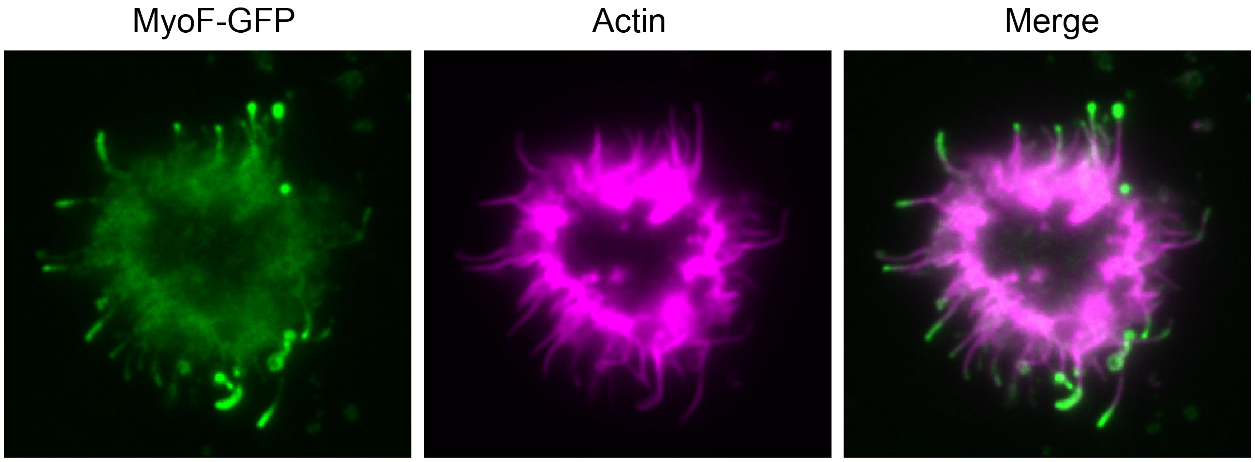

Fig. S5. Ectopic expression of MyoF-GFP in *Sf9* cells.

Epifluorescence microscopy images showing the localization of full-length MyoF (green) in *Sf9* cells expressing MyoF fused at its C-terminus with an mClover3 variant of GFP. Actin (magenta) is labeled with rhodamine-phalloidin.

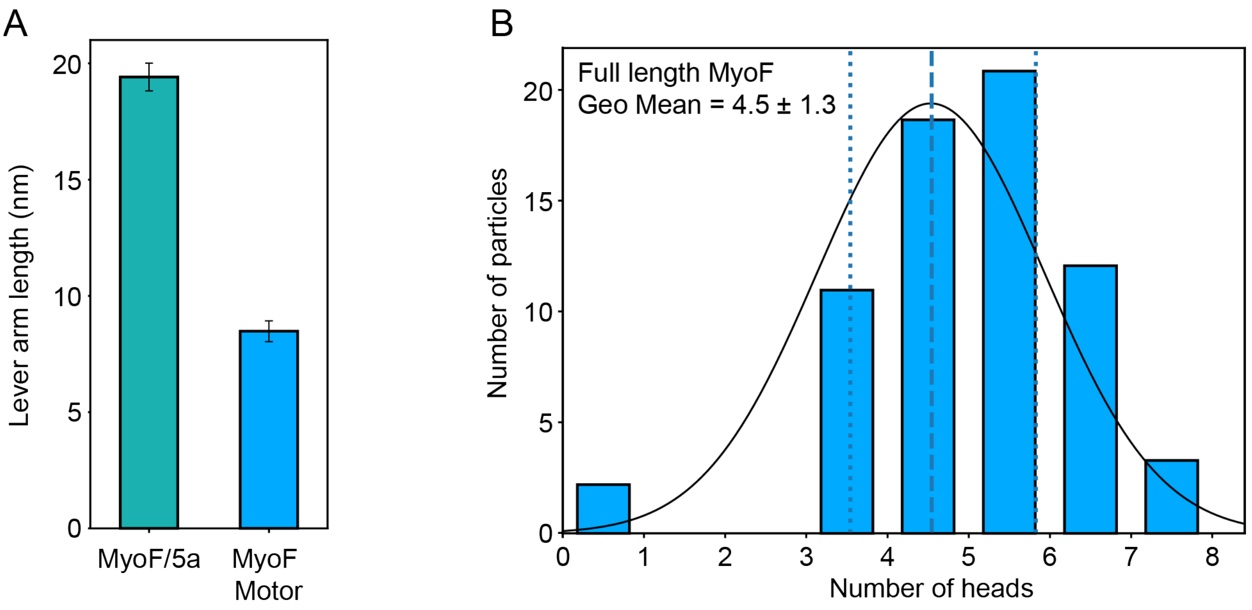

Fig. S6. Quantification of negatively stained electron microscopy images of the MyoF constructs.

(**A**) Average lever arm length ± SEM of negatively stained EM images of Myo5/MyoF chimera and MyoF Motor constructs (Myo5/MyoF: 19.4 ± 0.6 nm, n = 20; MyoF Motor: 8.6 ± 0.5 nm, n = 20). (**B**) Histogram of number of visible heads of negatively stained EM images full-length MyoF. Mean is calculated from the geometric mean ± standard deviation (n = 62).

Journal of Cell Science • Supplementary information

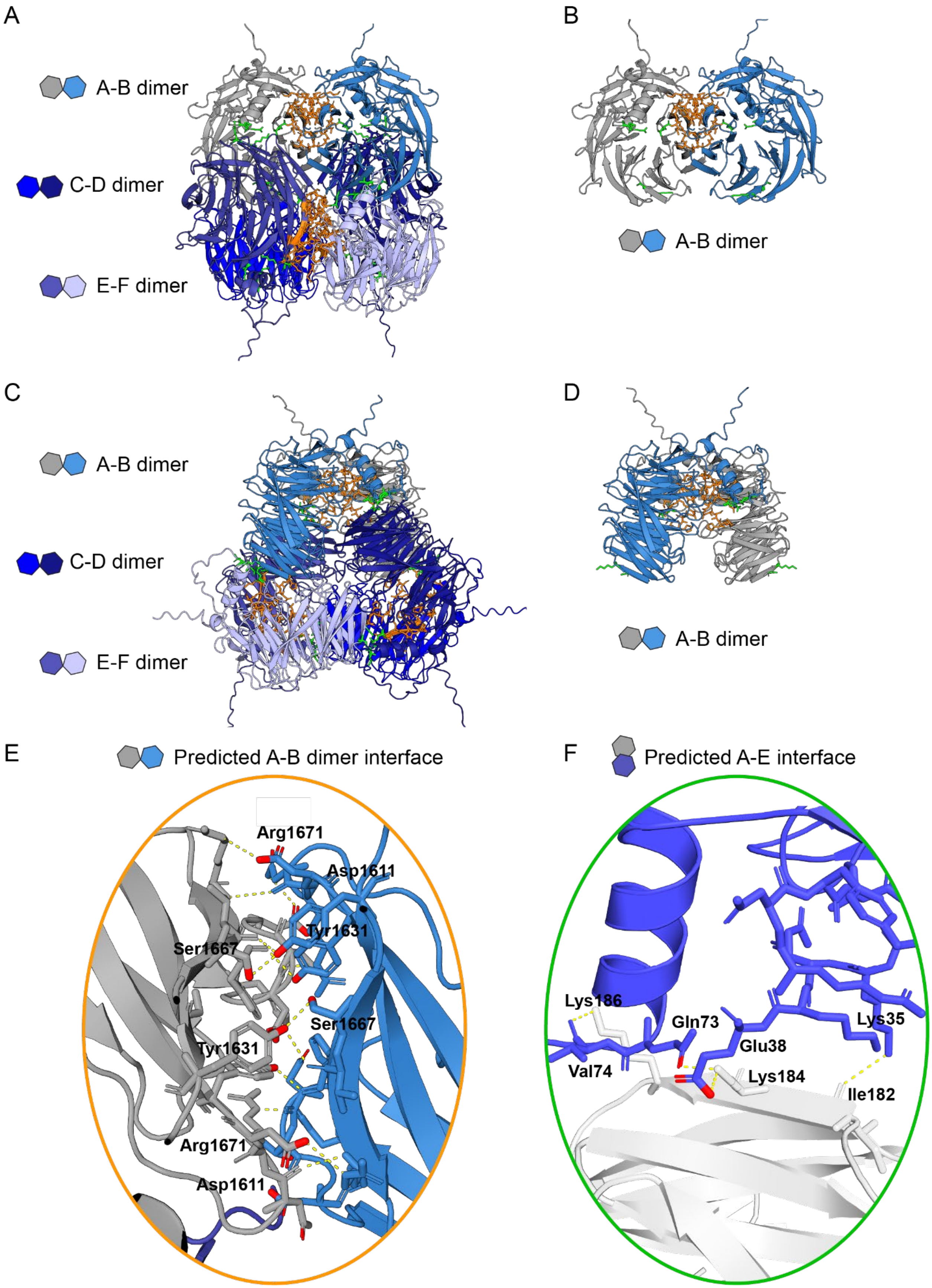

Journal of Cell Science • Supplementary information

Fig. S7. Structural prediction of MyoF WD40 in a hexameric arrangement using AlphaFold.

The WD40 domain of MyoF (amino acids M1572-V1953) was assembled into a hexameric oligomerization using AlphaFold. The resulting structure (**A**) is stabilized by an extensive hydrogen bonding network at three main interfaces (orange) between monomers annotated here as AB, CD, and EF and a second interface (green) between AC, AE, BD, and BF. The oligomeric structure predicts that the N-termini of individual WD40 domains are arranged as three adjacent pairs, consistent with a possible trimer of dimers arrangement of MyoF. (**B**) Same orientation as in (**A**), except only the AB dimer is shown. (**C**) The structure in (**A**) rotated 90 degrees about the y-axis. (**D**) Same orientation as in (**C**), except only the AB dimer is shown. (**E**) Each of the three dimer interfaces is stabilized by the same amino acid pairs. Thus, only one (AB) interface is shown. Asp1612, Try1613, Ser1667, and Arg1671 in chains B, D, and F form sidechain interactions with Arg1671, Ser1667, Try1631, and Asp1612 in chains A, C, and E respectively. Hydrogen bonding also occurs between Tyr1613 of chains B, D, and F with the carbonyl backbone of His1669 in chains A, C, and E. Tyr1613 and Tyr1631 in chains A, C, and E make additional interactions with the backbone of His1669 and Val1668 in B, D, and F. (**F**) Shows the interface between A and E which joins two pairs of dimers. Gln73 and Glu 38 in chain E make hydrogen bonding contacts with Lys184 in Chain A. Lys186 and Lys35 in Chain E binds the carbonyl backbone of Val74 and Ile182 in Chain A. Polar contacts are shown as yellow dashed lines.

Journal of Cell Science • Supplementary information

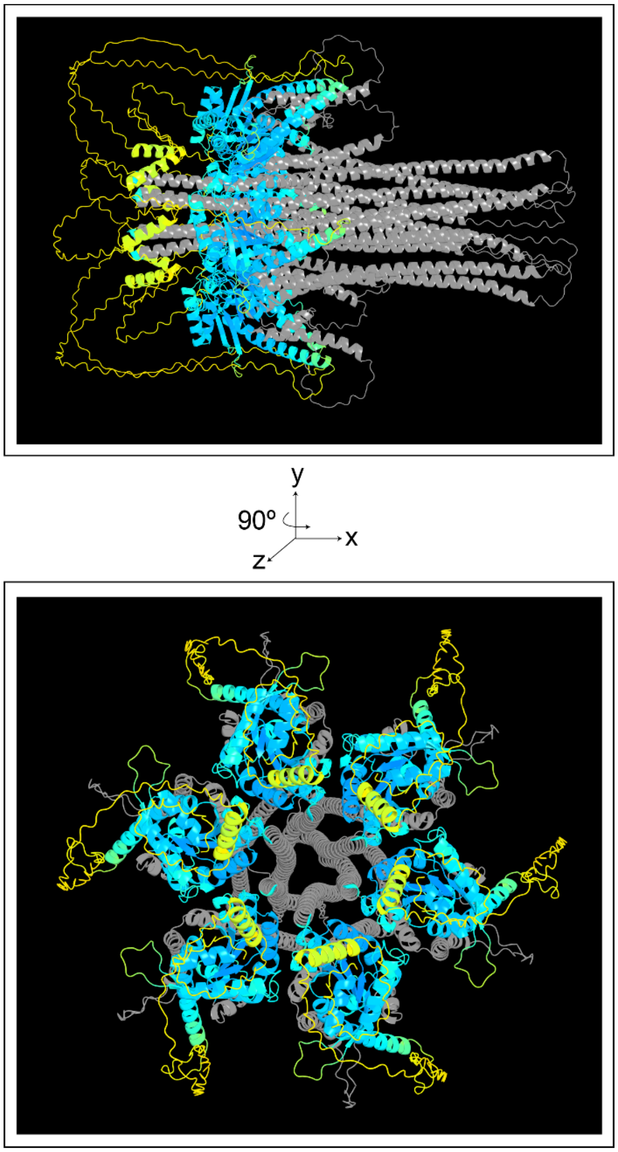

Fig. S8. A model of the MyoF structure between the coiled-coil and the WD40 domain associates with the alpha-helical coiled-coil region and is poorly defined.

AlphaFold prediction of a hexameric arrangement of the MyoF predicted coiled-coil sequence (residues E993 – W1251, grey) and mid region before the WD40 tail domain (residues I1252 – A1571). The mid region is shown associated with an alpha helical bundle and color coded by pLDDT (predicted Local Distance Difference Test), a measure of the model's confidence in the predicted local structure (red: <50, yellow 50-69, light blue 70-89, dark blue 90-100). The yellow region between P1465 to A1571 indicates low confidence. pTM and ipTM scores, a measure of the overall predicted fold, are 0.27 and 0.29 respectively, indicating low confidence in the overall predicted structure.

Journal of Cell Science • Supplementary information

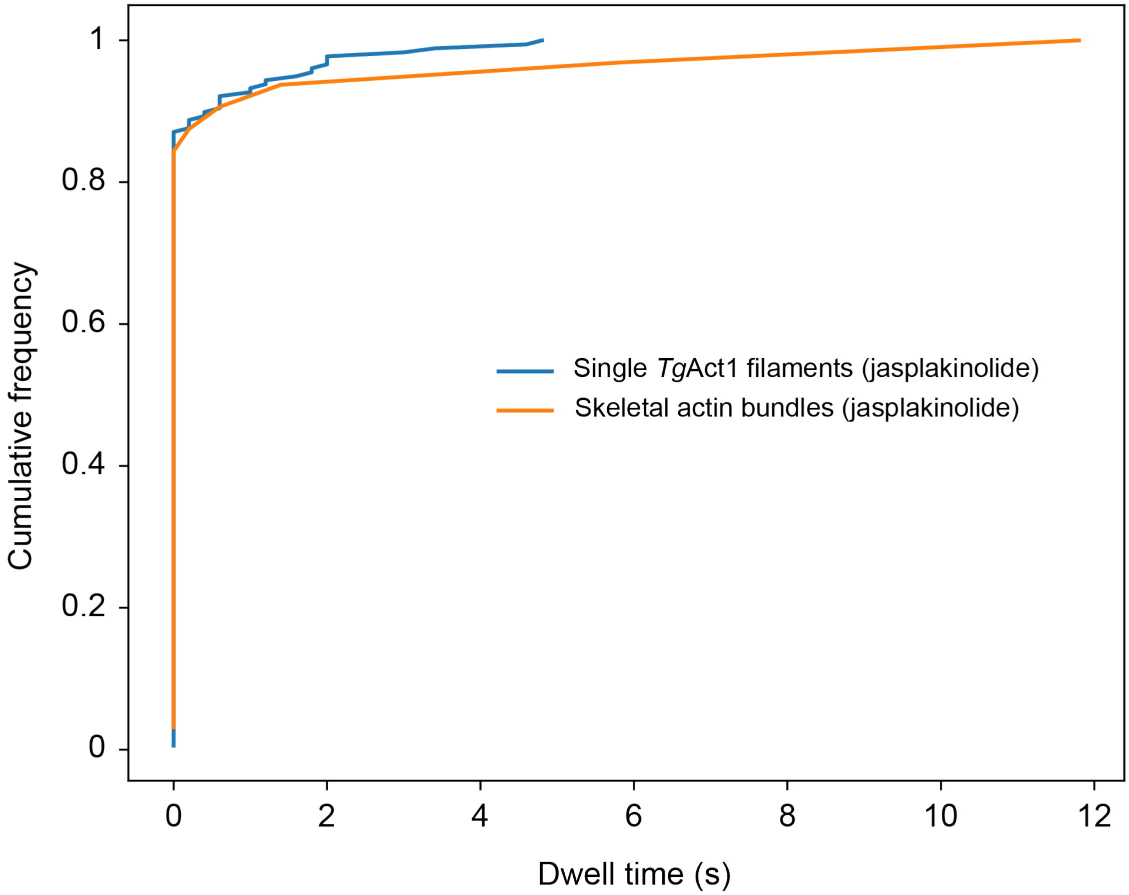

Fig. S9. Dwell time distribution of single molecules of full-length MyoF moving processively on *Tg*Act1 filaments versus skeletal actin bundles.

Distribution showing observed dwell times at the end of the (blue) single *Tg*Act1 filaments or (orange) skeletal actin bundles stabilized with jasplakinolide. *Tg*Act1 filaments (pause frequency, 13.4%; mean pause time (excluding zeros) ± SEM, 1.5 ± 0.26 seconds; n = 179) and skeletal actin bundles (pause frequency, 6.6%; mean pause time (excluding zeros) ± SEM, 4 ± 2.2 seconds; n = 33).

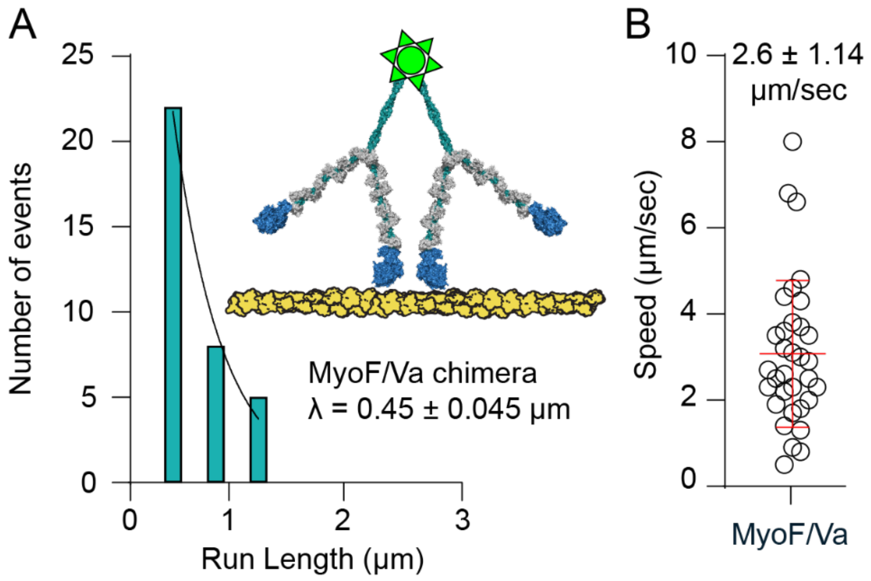

Fig. S10. Motility of small ensembles of MyoF/Va on skeletal vs. *Tg*Act1 actin filaments.

(**A**) Run length histogram of motor ensembles of the MyoF/Va chimeric construct bound to CamΔall linked through a Qdot on *T. gondii* actin (n = 33). (**B**) Speed distribution of MyoF/Va multiple motor motility on *T. gondii* actin. The mean speed is indicated with a red line. Error is in SD; n = 33.

Journal of Cell Science • Supplementary information

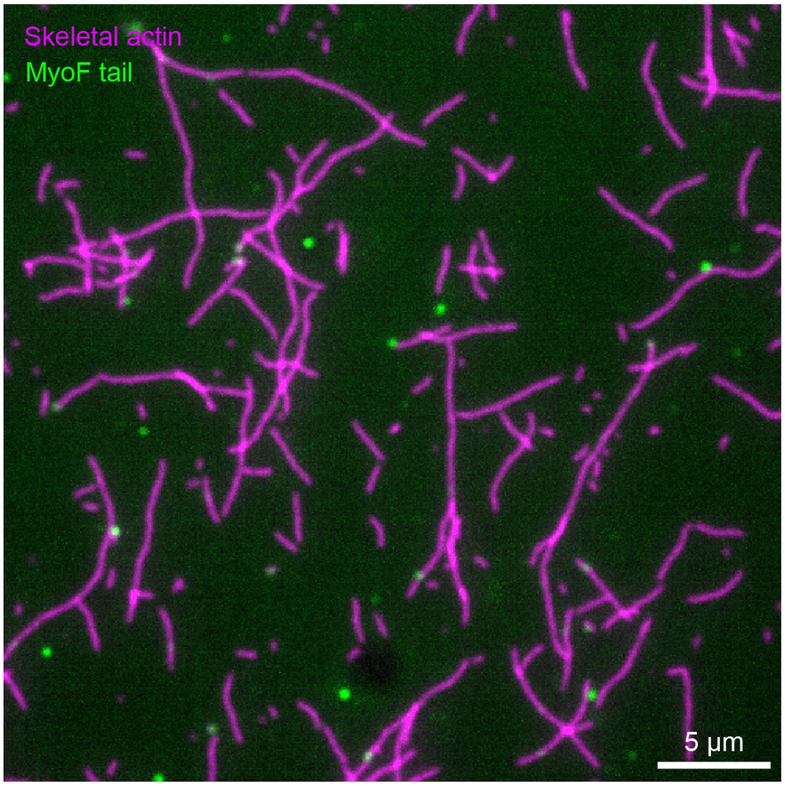

Fig. S11. The MyoF tail does not bind actin filaments directly.

Representative epifluorescence microscopy image showing that the MyoF tail labeled with Alexa Fluor 488 streptavidin (Green) does not bind directly to rhodamine-phalloidin stabilized skeletal actin filaments (magenta) under low ionic strength conditions (10 mM KOAc).

Journal of Cell Science • Supplementary information

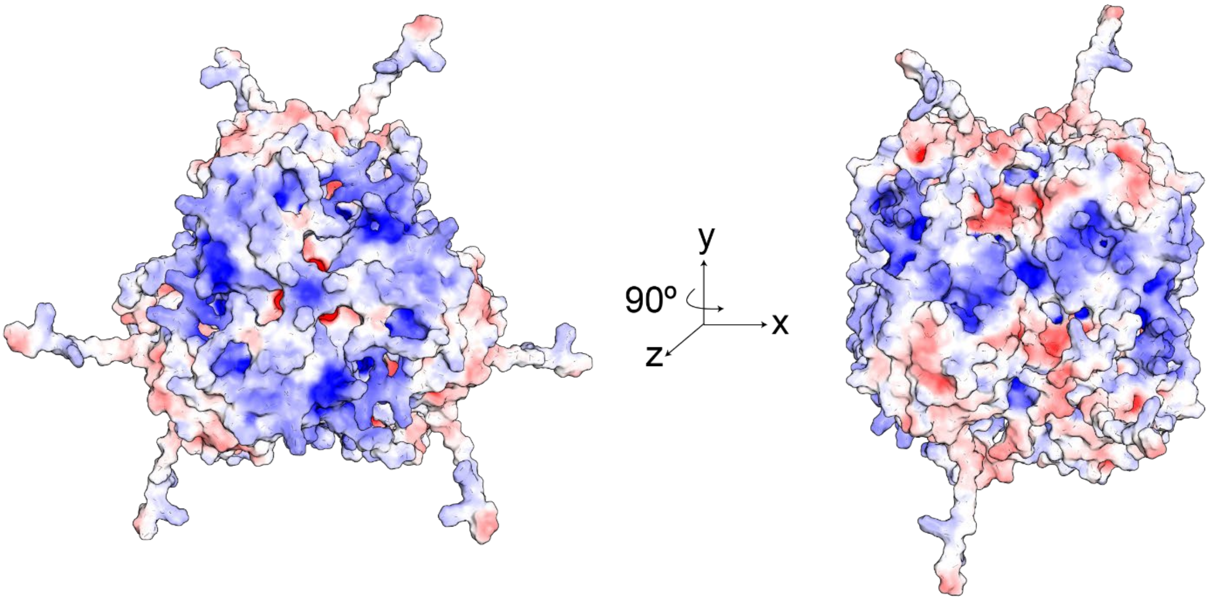

Fig. S12. Electrostatic surface potential of the hexameric MyoF WD40 domain.

Electrostatic surface potential of the AlphaFold-predicted hexameric WD40 assembly rendered using the APBS electrostatics plugin in PyMOL. Surfaces are colored according to electrostatic potential (red, negative; white, neutral; blue, positive). Left, side view of the hexamer with β-propeller domains oriented laterally. This orientation reveals a broad, continuous electropositive surface spanning multiple WD40 domains. Right, the same assembly rotated 90° to display a face-on view of the β-propeller arrangement. In this orientation, a longitudinal strip of electropositive potential extends across adjacent propellers, forming a continuous basic surface along one axis of the complex.

Table S1. Summary of MyoF-interacting proteins from *T. gondii* cell lysates

IP replicate 1_accession number	Annotation	# peptides
TGME49_278870	myosin F	118
TGME49_239400	inner membrane complex protein IMC28	13
TGME49_227870	Tim10/DDP family zinc finger superfamily protein	3
TGME49_257680	myosin light chain MLC1	2
TGME49_203260	cell cycle checkpoint protein RAD17, putative	1
TGME49_203980	hypothetical protein	1
TGME49_209910	histone H2Bv	1
TGME49_221620	beta-tubulin, putative	1
TGME49_223750	hypothetical protein	1
TGME49_223940	gliding-associated protein GAP45	1
TGME49_228660	Sec7 domain-containing protein	1
TGME49_228690	phosphatidylinositol 3- and 4-kinase	1
TGME49_231630	alveolin domain containing intermediate filament IMC4	1
TGME49_232280	hypothetical protein	1
TGME49_233460	SAG-related sequence SRS29B	1
TGME49_254610	Tim10/DDP family zinc finger superfamily protein	1
TGME49_259080	hypothetical protein	1
TGME49_260790	RAP domain-containing protein	1
TGME49_261250	histone H2A1	1
TGME49_264485	AP2 domain transcription factor AP2IX-3	1
TGME49_291050	serine/threonine-protein kinase KIN	1
TGME49_293590	3-oxoacyl-acyl-carrier protein synthase I/II, putative	1
TGME49_308090	rhoptry protein ROP5	1
TGME49_309885	hypothetical protein	1
TGME49_310010	rhoptry neck protein RON1	1

Journal of Cell Science • Supplementary information

IP replicate 1_accession number	Annotation	# peptides
TGME49_311240	DnaJ family chaperone J1	1
TGME49_312520	tRNA dimethylallyltransferase	1
TGME49_313140	isocitrate dehydrogenase 2	1
TGME49_316760	hypothetical protein	1

IP replicate 2_accession number	Annotation	# peptides
TGME49_278870	myosin F	302
TGME49_249240	calmodulin, putative	45
TGME49_290870	patched family protein	22
TGME49_236650	DEAD (Asp-Glu-Ala-Asp) box polypeptide 17	21
TGME49_239400	inner membrane complex protein IMC28	16
TGME49_266960	beta tubulin	12
TGME49_269190	glyceraldehyde-3-phosphate dehydrogenase GAPDH2	11
TGME49_273760	heat shock protein HSP70	10
TGME49_237180	hypothetical protein	10
TGME49_233460	SAG-related sequence SRS29B	9
TGME49_215775	rhoptry protein ROP8	8
TGME49_310010	rhoptry neck protein RON1	8
TGME49_232410	thioredoxin-like protein 1	8
TGME49_311720	chaperonin protein BiP	7
TGME49_231630	alveolin domain containing intermediate filament IMC4	7
TGME49_235470	myosin A	7
TGME49_316400	alpha tubulin TUBA1	6
TGME49_219320	gliding-associated protein GAP50	6
TGME49_231640	alveolin domain containing intermediate filament IMC1	6
TGME49_239260	histone H4	6
TGME49_257680	myosin light chain MLC1	6

Journal of Cell Science • Supplementary information

IP replicate 1_accession number	Annotation	# peptides
TGME49_236540	RNA recognition motif-containing protein	6
TGME49_286420	elongation factor 1-alpha (EF-1-ALPHA), putative	5
TGME49_213392	surface antigen repeat-containing protein	5
TGME49_319560	microneme protein MIC3	4
TGME49_258870	cyst wall protein CST7	4
TGME49_258580	rhoptry protein ROP17	4
TGME49_305160	histone H2Ba	4
TGME49_223920	rhoptry neck protein RON3	4
TGME49_288650	dense granule protein GRA12	4
TGME49_313380	IMC localizing protein ILP1	4
TGME49_250340	centrin 2	4
TGME49_300200	histone H2AZ	4
TGME49_289750	ribosomal-ubiquitin protein RPL40	3
TGME49_209910	histone H2Bv	3
TGME49_261580	histone H2AX	3
TGME49_311240	DnaJ family chaperone J1	3
TGME49_263090	14-3-3 protein	3
TGME49_249900	adenine nucleotide translocator	3
TGME49_308090	rhoptry protein ROP5	3
TGME49_232940	heat shock protein HSP20	3
TGME49_203310	dense granule protein GRA7	2
TGME49_216000	alveolin domain containing intermediate filament IMC3	3
TGME49_279100	mitochondrial association factor 1a	3
TGME49_295110	rhoptry protein ROP7	3
TGME49_218260	histone H3.3	2
TGME49_271050	SAG-related sequence SRS34A	2

Journal of Cell Science • Supplementary information

IP replicate 1_accession number	Annotation	# peptides
TGME49_295125	rhoptry protein ROP4	2
TGME49_209030	actin ACT1	2
TGME49_262620	RNA recognition motif-containing protein	2
TGME49_211680	protein disulfide isomerase	2
TGME49_248700	alveolin domain containing intermediate filament IMC12	2
TGME49_243950	prohibitin, putative	2
TGME49_227280	dense granule protein GRA3	2
TGME49_261950	ATP synthase beta subunit ATP-B	2
TGME49_205658	F5/8 type C domain-containing protein	2
TGME49_201780	microneme protein MIC2	2
TGME49_231160	hypothetical protein	2
TGME49_219270	glideosome-associated protein with multiple-membrane spans GAPM2A	2
TGME49_207040	hypothetical protein	2
TGME49_220270	alveolin domain containing intermediate filament IMC6	2
TGME49_235402	CorA family Mg2+ transporter protein	2
TGME49_293590	3-oxoacyl-acyl-carrier protein synthase I/II, putative	2
TGME49_209485	hypothetical protein, conserved	1
TGME49_207800	hypothetical protein	1
TGME49_229480	calcium binding protein precursor, putative	1
TGME49_215980	hypothetical protein	1
TGME49_215980	hypothetical protein	1
TGME49_217340	hypothetical protein	1
TGME49_306030	glutathione S-transferase 2	1
TGME49_306920	HOOK interacting protein HIP	1
TGME49_258410	photosensitized INA-labeled protein PHIL1	1

Journal of Cell Science • Supplementary information

IP replicate 1_accession number	Annotation	# peptides
TGME49_208030	microneme protein MIC4	1
TGME49_235930	domain K- type RNA binding proteins family protein	1
TGME49_257540	hypothetical protein	1
TGME49_269980	preprotein translocase Sec61, putative	1
TGME49_249480	co-chaperone UNC	1
TGME49_288380	heat shock protein HSP90	1
TGME49_310460	ras-related protein RAB6	1
TGME49_219660	hypothetical protein	1
TGME49_313140	isocitrate dehydrogenase 2	1
TGME49_305630	F-box domain-containing protein	1
TGME49_306460	bromodomain-containing protein BDP4	1
TGME49_227080	PCIF1 WW domain-containing protein	1
TGME49_229150	transporter, major facilitator family protein	1
TGME49_204400	ATPase synthase subunit alpha, putative	1
TGME49_272490	protoporphyrinogen oxidase	1
TGME49_278130	basal complex component BCC11	1
TGME49_259630	inner membrane complex protein IMC27	1
TGME49_309885	hypothetical protein	1
TGME49_224850	polyadenylate-binding protein PABC	1
TGME49_270250	dense granule protein GRA1	1
TGME49_273445	iron-sulfur cluster assembly protein SufD	1
TGME49_230160	RNA-binding protein, putative	1

IP replicate 3_accession number	Annotation	# peptides
TGME49_278870	myosin F	162
TGME49_249240	calmodulin, putative	21
TGME49_290870	patched family protein	11

Journal of Cell Science • Supplementary information

IP replicate 1_accession number	Annotation	# peptides
TGME49_236650	DEAD (Asp-Glu-Ala-Asp) box polypeptide 17	9
TGME49_239400	inner membrane complex protein IMC28	5
TGME49_237180	hypothetical protein	5
TGME49_233460	SAG-related sequence SRS29B	4
TGME49_215775	rhoptry protein ROP8	4
TGME49_310010	rhoptry neck protein RON1	4
TGME49_286420	elongation factor 1-alpha (EF-1-ALPHA), putative	4
TGME49_226790	ABC transporter, ATP-binding domain-containing protein	2
TGME49_305240	XPA binding protein 2 family protein	2
TGME49_223985	protein phosphatase PPM11B, putative	2
TGME49_292320	queuine tRNA-ribosyltransferase, putative	2
TGME49_290620	centrosomal protein CEP250L1	2
TGME49_316400	alpha tubulin TUBA1	1
TGME49_219320	gliding-associated protein GAP50	1
TGME49_258580	rhoptry protein ROP17	1
TGME49_319560	microneme protein MIC3	1
TGME49_258870	cyst wall protein CST7	1
TGME49_289750	ribosomal-ubiquitin protein RPL40	1
TGME49_203310	dense granule protein GRA7	1
TGME49_271050	SAG-related sequence SRS34A	1
TGME49_218260	histone H3.3	1
TGME49_207040	hypothetical protein	1
TGME49_235402	CorA family Mg2+ transporter protein	1
TGME49_227620	dense granule protein GRA2	1
TGME49_234505	phenylalanyl-tRNA synthetase alpha chain A, putative	1
TGME49_216170	cysteine desulfurase NFS2	1

Journal of Cell Science • Supplementary information

IP replicate 1_accession number	Annotation	# peptides
TGME49_248160	ATP-dependent DNA helicase 2 subunit KU70	1
TGME49_212045	hypothetical protein, conserved	1
TGME49_266880	dihydrouridine synthase, putative	1
TGME49_255700	hypothetical protein	1
TGME49_205470	translation elongation factor 2 family protein, putative	1
TGME49_210700	VPS13 domain-containing protein	1
TGME49_222930	hypothetical protein	1
TGME49_223080	hypothetical protein	1
TGME49_277950	lipase	1
TGME49_237460	DER1-like protein	1
TGME49_227010	rhoptry kinase family protein ROP30	1
TGME49_286450	dense granule protein GRA5	1
TGME49_244910	MIZ/SP-RING zinc finger domain-containing protein	1
TGME49_240970	hypothetical protein	1

Present in at least 2 of 3 datasets	Annotation	Total # peptides
TGME49_278870	myosin F	582
TGME49_249240	calmodulin, putative	66
TGME49_239400	inner membrane complex protein IMC28	34
TGME49_290870	patched family protein	33
TGME49_236650	DEAD (Asp-Glu-Ala-Asp) box polypeptide 17	30
TGME49_237180	hypothetical protein	15
TGME49_233460	SAG-related sequence SRS29B	14
TGME49_310010	rhoptry neck protein RON1	13
TGME49_215775	rhoptry protein ROP8	12
TGME49_286420	elongation factor 1-alpha (EF-1-ALPHA), putative	9
TGME49_257680	myosin light chain MLC1	8

Journal of Cell Science • Supplementary information

IP replicate 1_accession number	Annotation	# peptides
TGME49_231630	alveolin domain containing intermediate filament IMC4	8
TGME49_316400	alpha tubulin TUBA1	7
TGME49_219320	gliding-associated protein GAP50	7
TGME49_258580	rhoptry protein ROP17	5
TGME49_319560	microneme protein MIC3	5
TGME49_258870	cyst wall protein CST7	5
TGME49_209910	histone H2Bv	4
TGME49_289750	ribosomal-ubiquitin protein RPL40	4
TGME49_308090	rhoptry protein ROP5	4
TGME49_311240	DnaJ family chaperone J1	4
TGME49_293590	3-oxoacyl-acyl-carrier protein synthase I/II, putative	3
TGME49_203310	dense granule protein GRA7	3
TGME49_271050	SAG-related sequence SRS34A	3
TGME49_218260	histone H3.3	3
TGME49_207040	hypothetical protein	3
TGME49_235402	CorA family Mg2+ transporter protein	3
TGME49_309885	hypothetical protein	2
TGME49_313140	isocitrate dehydrogenase 2	2

Journal of Cell Science • Supplementary information

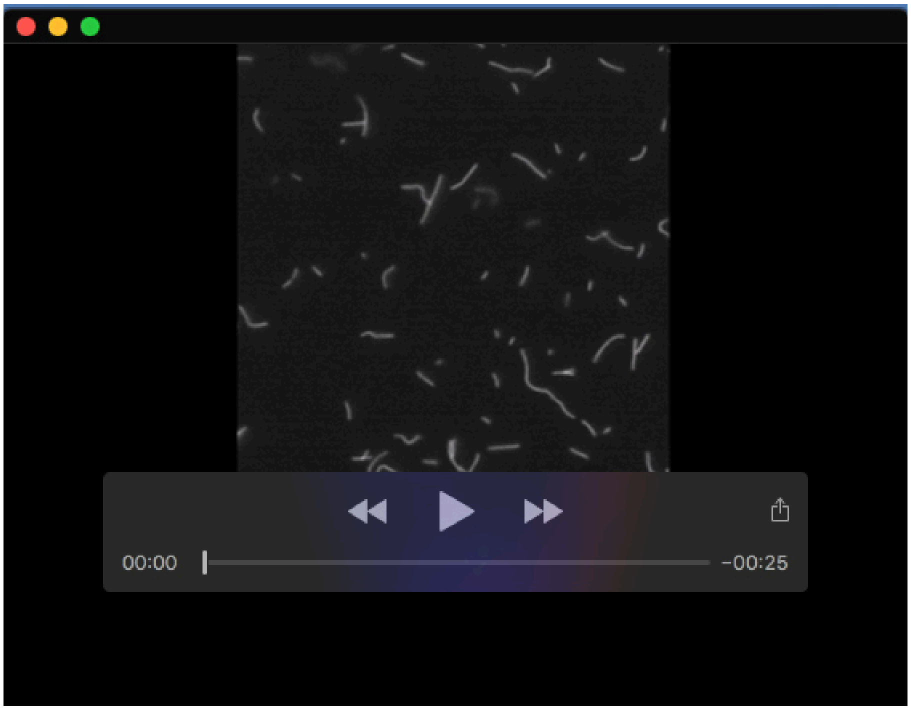

Movie 1. Gliding filament *in vitro* motility of MyoF-motor co-purified with *Tg*Cam and *Tg*Mlc1. Frames acquired at 1 sec intervals, 37°C. 15x playback, Image width 32.5 μm.

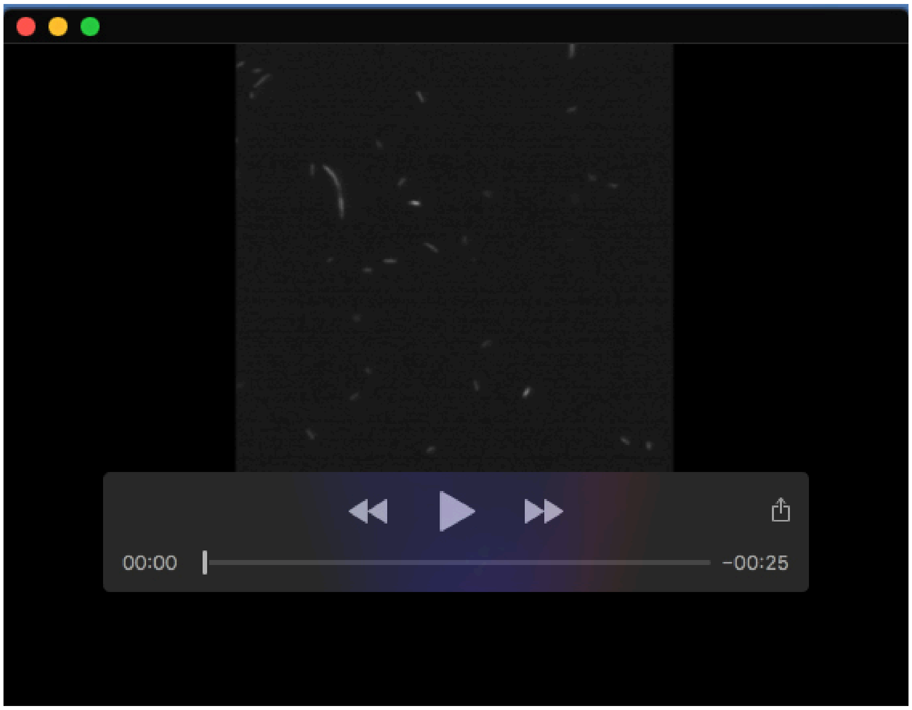

Movie 2. Gliding filament *in vitro* motility of MyoF-motor co-purified with *Tg*Cam. Frames acquired at 1 sec intervals, 37°C. 15x playback, Image width 32.5 μm.

Journal of Cell Science • Supplementary information

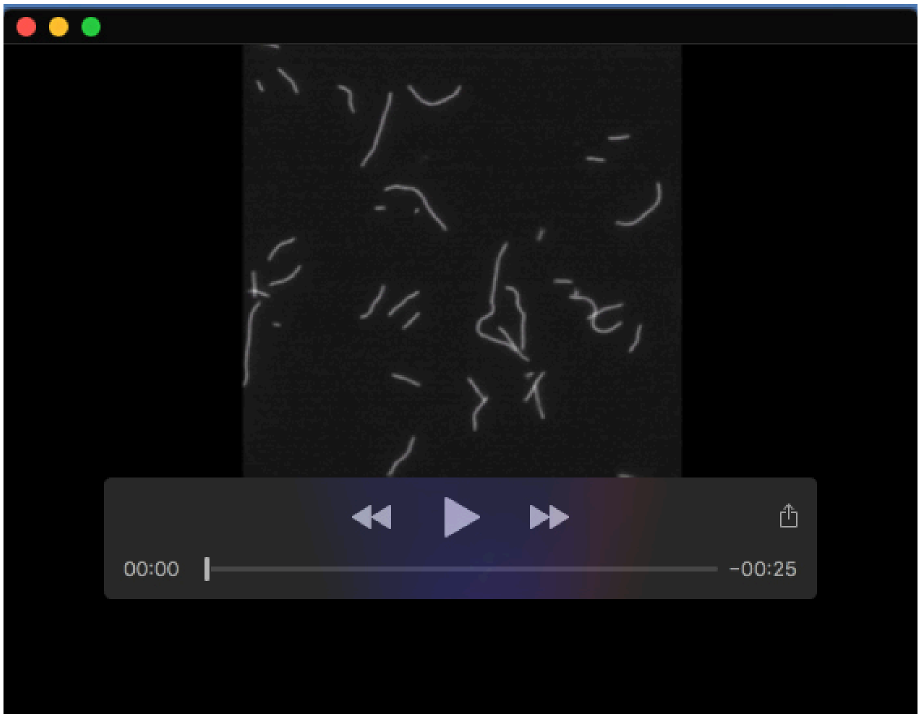

Movie 3. Gliding filament *in vitro* motility of MyoF-motor co-purified with *Tg*Mlc1. Frames acquired at 1 sec intervals, 37°C. 15x playback, Image width 32.5 μm.

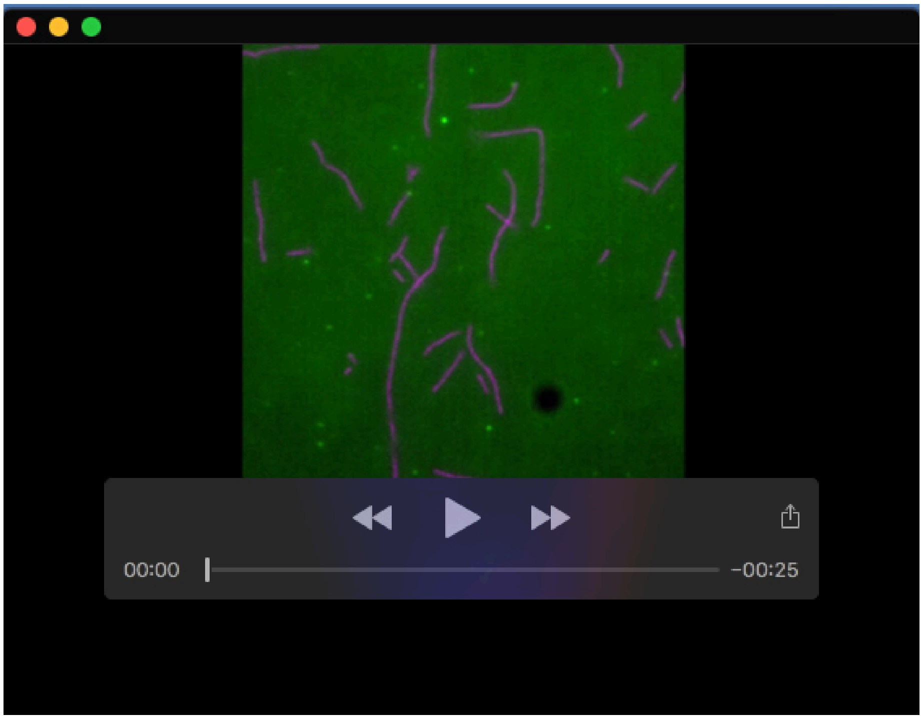

Movie 4. Single molecule motility of MyoF-motor co-purified with *Tg*Cam and *Tg*Mlc1 bound to a streptavidin conjugated Qdot 655 (green) on skeletal actin stabilized with Alexa 488 phalloidin (magenta). **No events were observed**. Frames acquired at 300 ms intervals, 37°C. 9x playback, Image width 32.5 μm.

Journal of Cell Science • Supplementary information

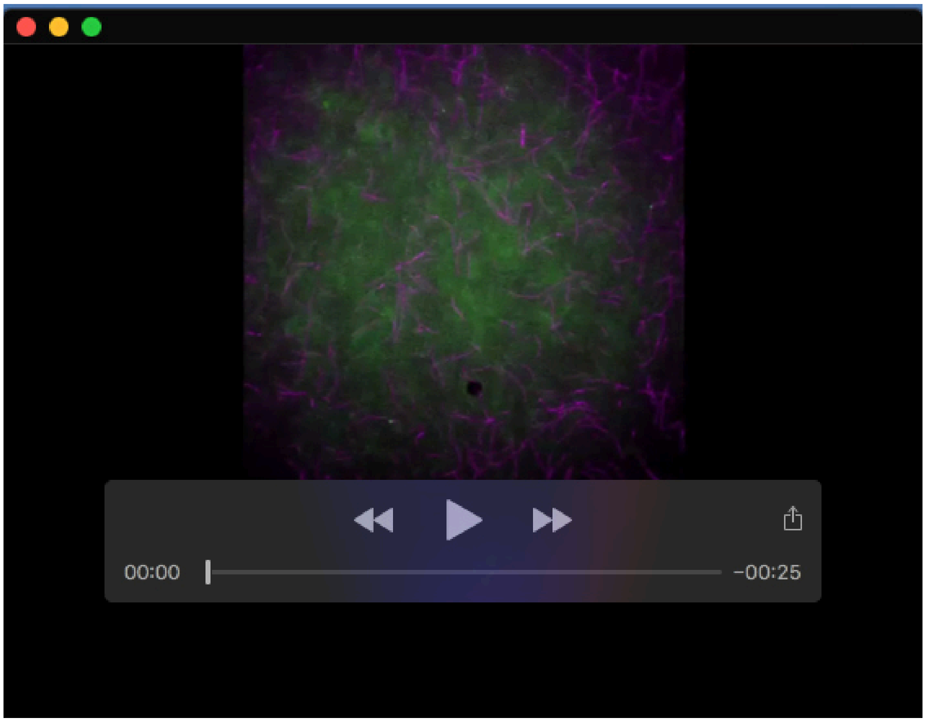

Movie 5. Single molecule motility of MyoF-motor co-purified with *Tg*Cam bound to a streptavidin conjugated Qdot 655 (green) on skeletal actin stabilized with Alexa 488 phalloidin (magenta). **No events were observed**. Frames acquired at 200 ms intervals, 37°C. 6x playback, Image width 66.6 µm.

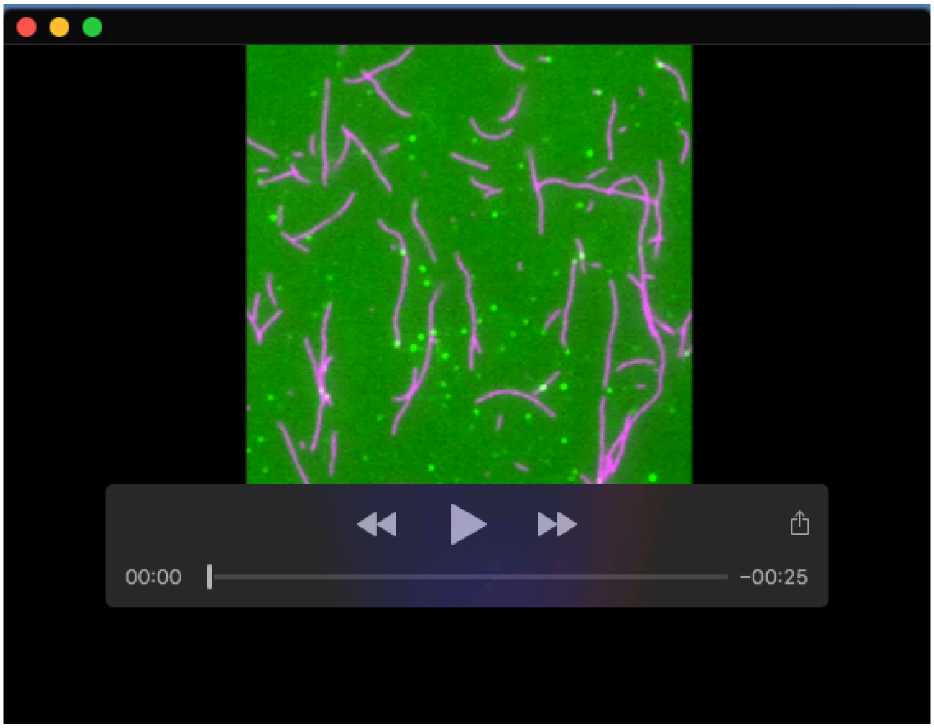

Movie 6. Movement of multiple motors of MyoF-motor co-purified with *Tg*Cam and *Tg*Mlc1 bound to a streptavidin conjugated Qdot 655 (green) on skeletal actin filaments (magenta). Frames acquired at 300 ms intervals, 37°C. 4.5x playback, Image width 32.5 µm.

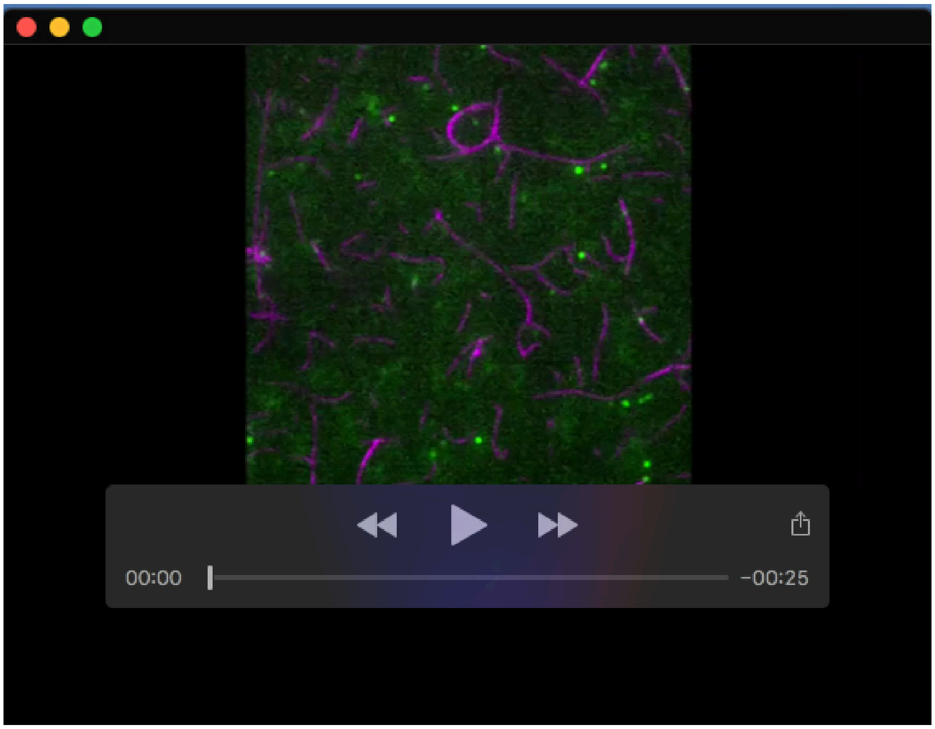

Movie 7. Movement of multiple motors of MyoF-motor co-purified with *Tg*Cam bound to a streptavidin conjugated Qdot 655 (green) on skeletal actin stabilized with Alexa 488 phalloidin (magenta). Frames acquired at 50 ms intervals, 37°C. 1x playback, Image width 32.5 µm.

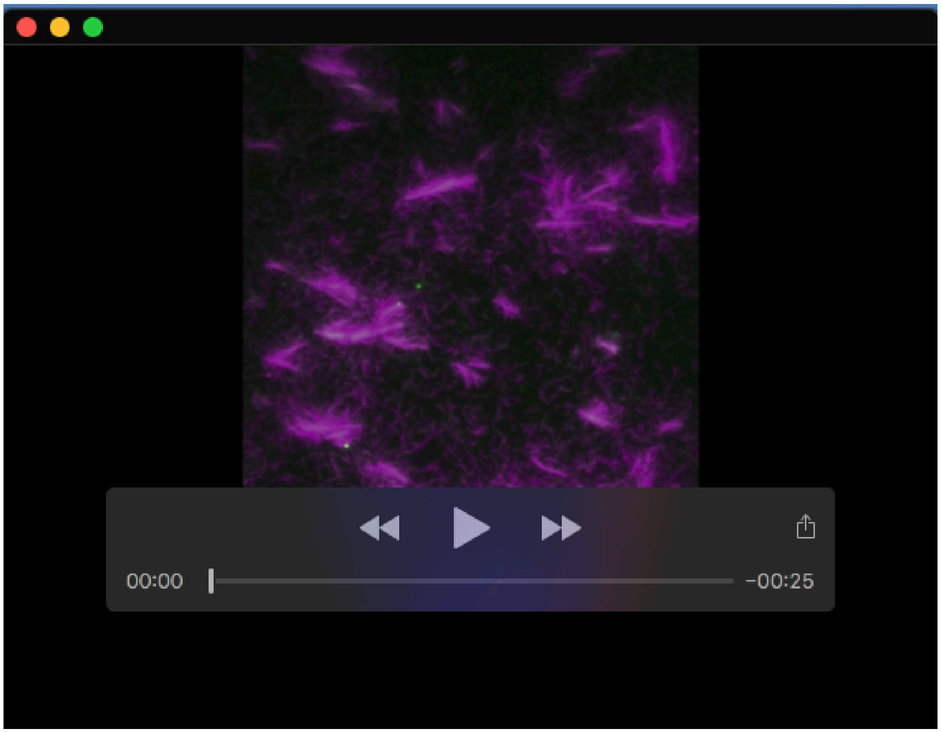

Movie 8. Single molecule motility of MyoF-motor co-purified with *Tg*Cam bound to a streptavidin conjugated Qdot 655 (green) on skeletal actin stabilized with Alexa 488 phalloidin bundled with the addition of a 1:1.1 molar ratio of fascin (magenta). **No events were observed**. Images were captured using TIRF microscopy. Frames acquired at 50 ms intervals, 23°C. 1x playback, Image width 55 µm.

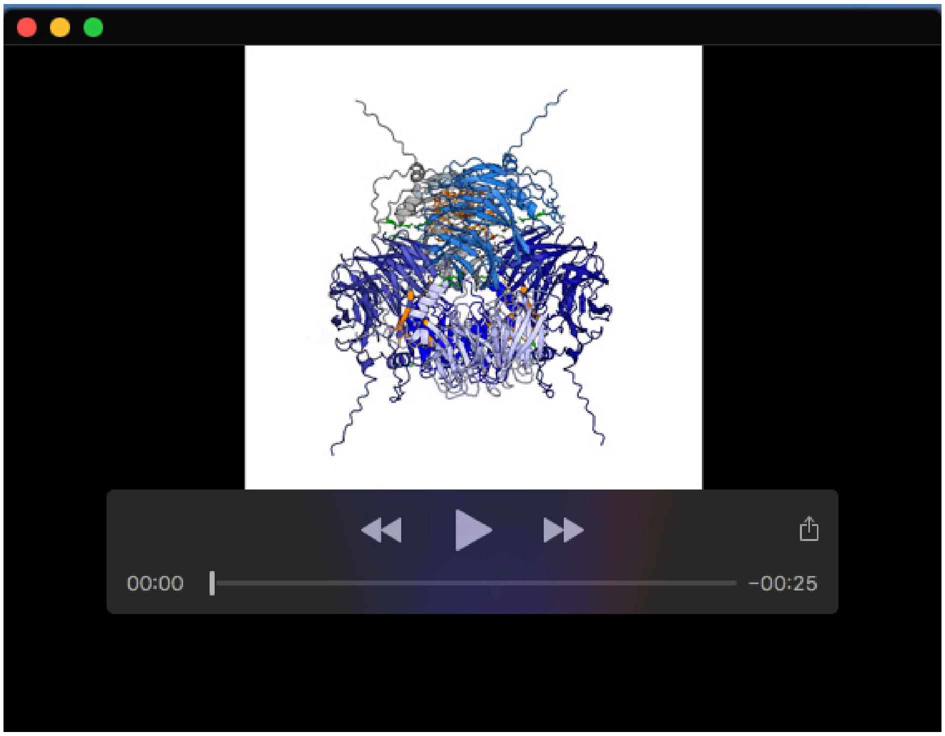

Movie 9. AlphaFold predicted structure of the hexameric MyoF WD40 tail domain. Residues that stabilize the dimeric interface are shown in orange and those that stabilize a trimeric organization of WD40 dimers are in green.

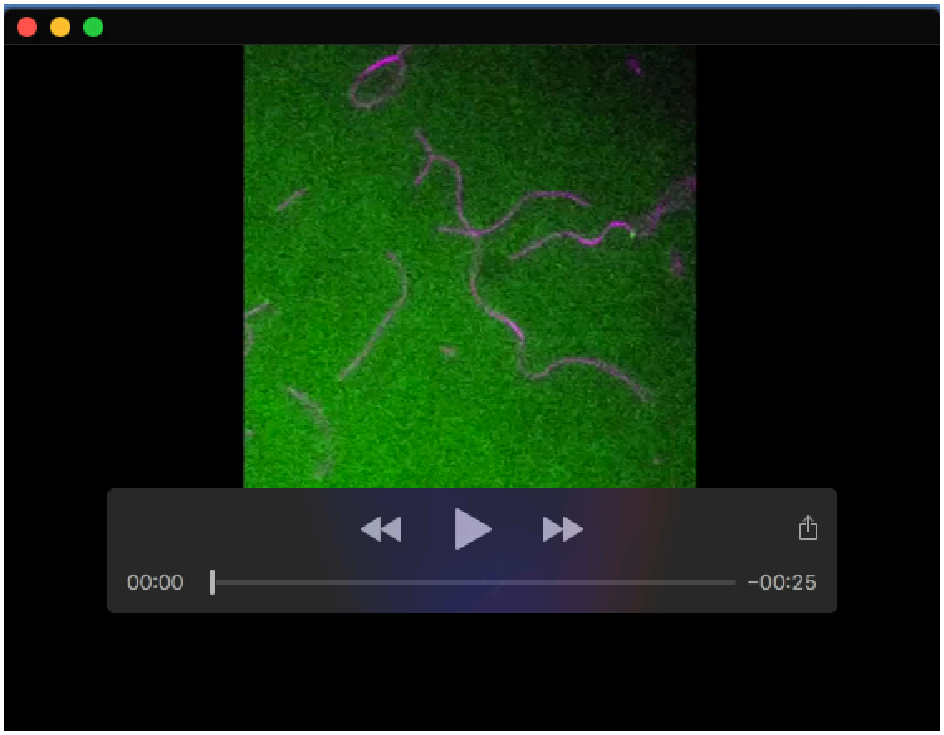

Movie 10. Single molecule motility of full-length MyoF co-purified with *Tg*Cam bound to a streptavidin conjugated Alexa Fluor 647 (green) on skeletal actin stabilized with Alexa 488 phalloidin. **No events were observed**. Frames acquired at 200 ms intervals, 37°C. 3x playback, Image width 32.5 μm.

Journal of Cell Science • Supplementary information

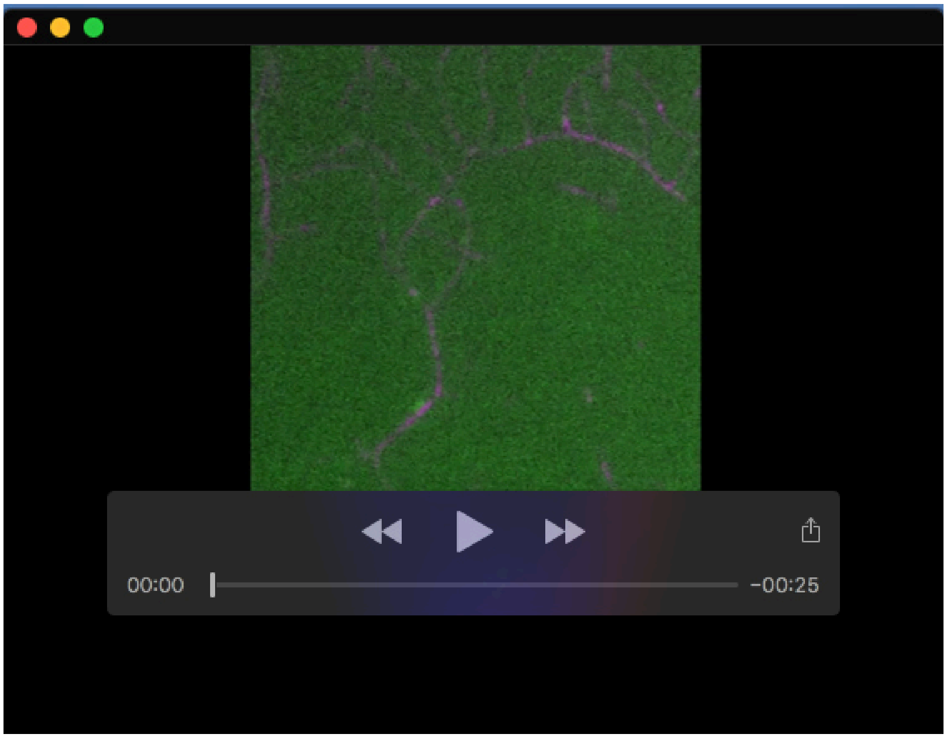

Movie 11. Single molecule motility of full-length MyoF co-purified with *Tg*Cam bound to a streptavidin conjugated Alexa Fluor 647 (green) on *Tg*Act1 actin filaments stabilized with jasplakinolide and visualized with chromobody fused to EmeraldFP (magenta). Frames acquired at 200 ms intervals, 37°C. 2x playback, Image width 25.1 μm.

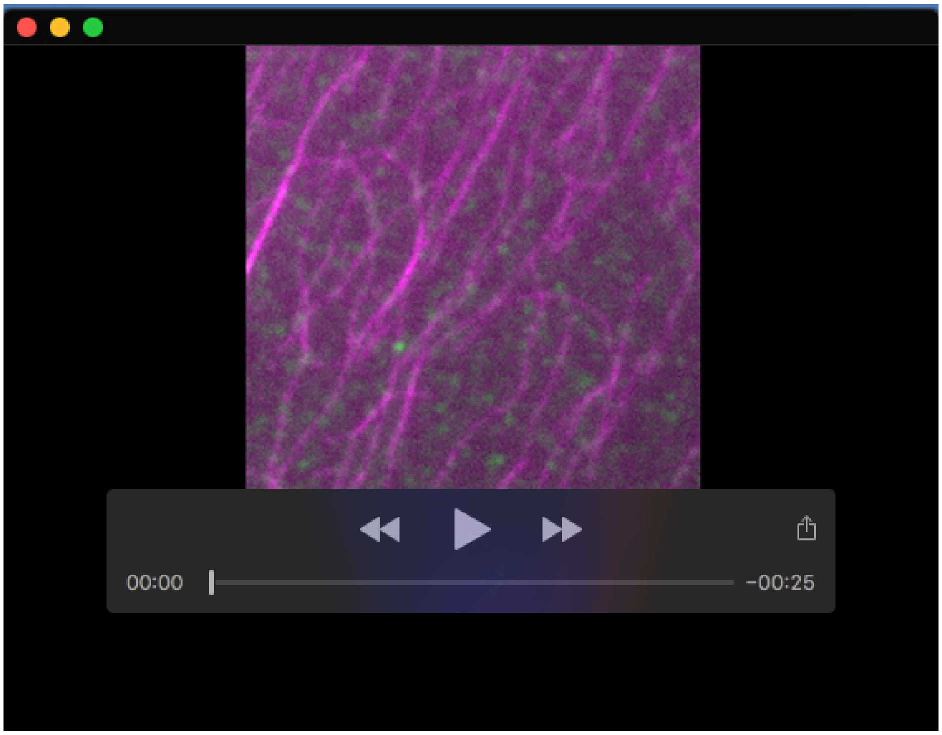

Movie 12. Single molecule motility of full-length MyoF co-purified with *Tg*Cam bound to a streptavidin conjugated Alexa Fluor 647 (green) on unstabilized *Tg*Act1 actin filaments and visualized with chromobody fused to EmeraldFP (magenta). Frames acquired at 1 s intervals, 37°C. 4x playback, Image width 15 μm.

Journal of Cell Science • Supplementary information

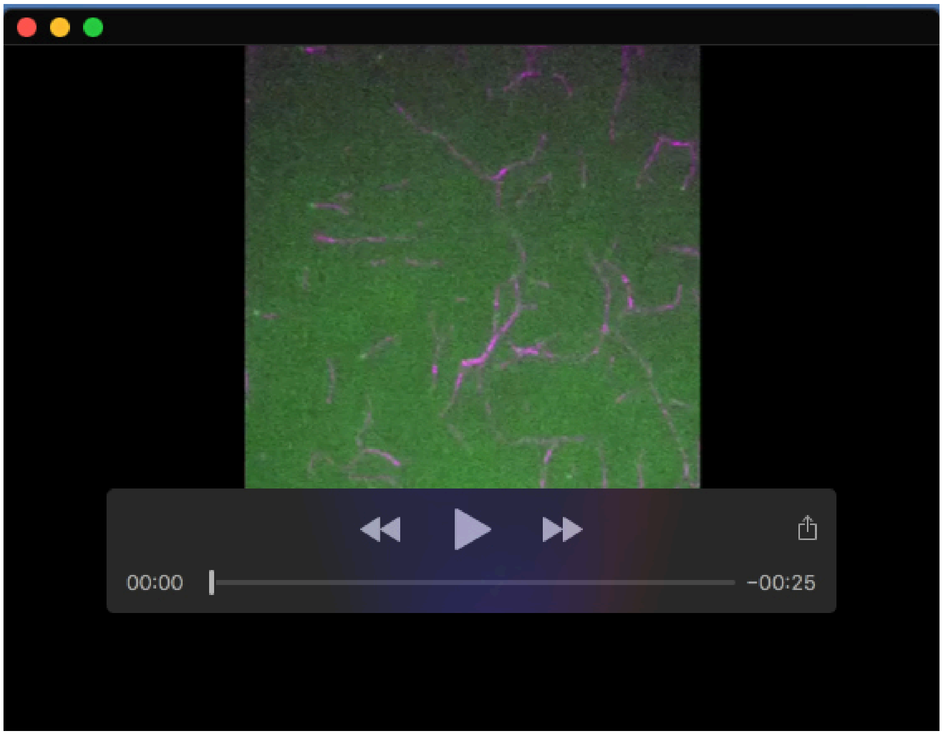

Movie 13. Single molecule motility of full-length MyoF co-purified with *Tg*Cam and *Tg*Mlc1 bound to a streptavidin conjugated Alexa Fluor 647 (green) on *Tg*Act1 actin filaments stabilized with jasplakinolide and visualized with chromobody fused to EmeraldFP (magenta). **No events were observed**. Frames acquired at 200 ms intervals, 37°C. 3x playback, Image width 32.5 µm.

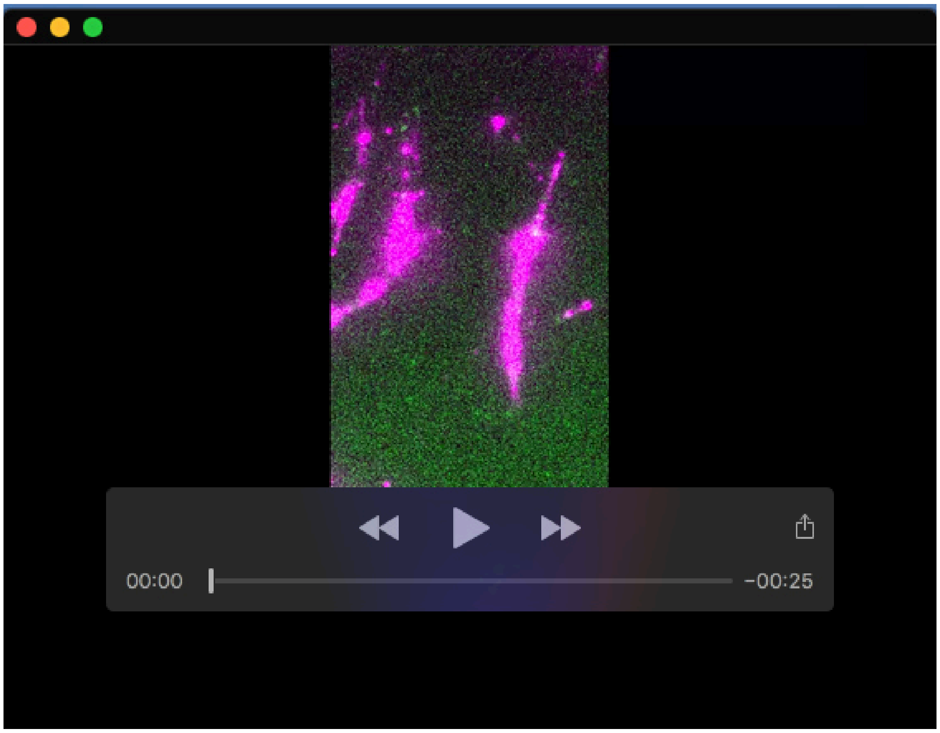

Movie 14. Movement of full-length MyoF co-purified with *Tg*Cam labeled with streptavidin conjugated Alexa Fluor 647 (green) on skeletal actin fascin bundles stabilized with Alexa 488 phalloidin (magenta). Frames acquired at 200 ms intervals, 37°C. 6x playback, Image width 17 µm.

Journal of Cell Science • Supplementary information

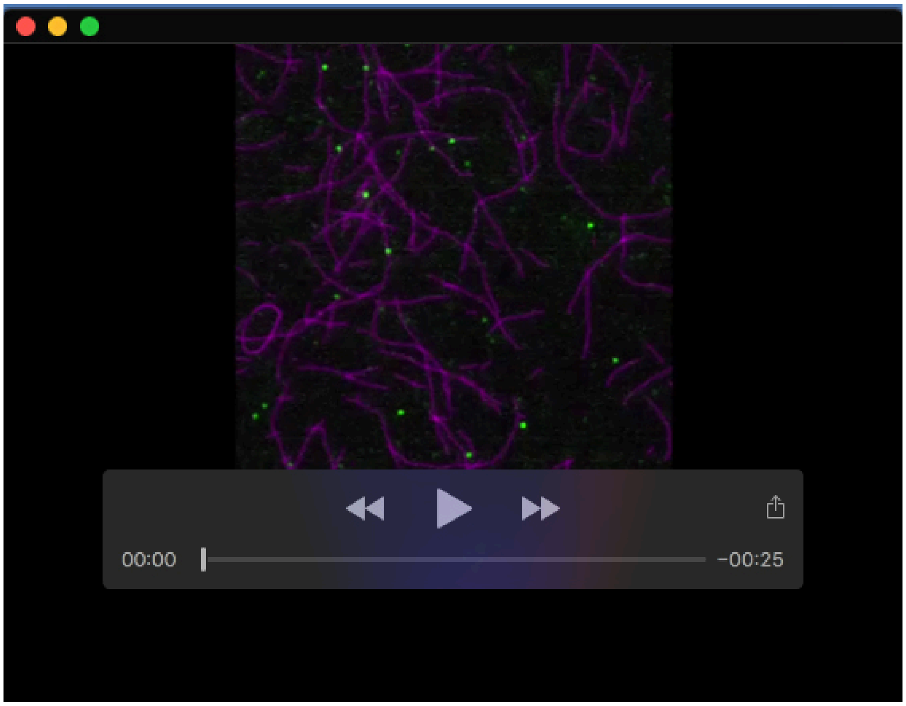

Movie 15. Movement of multiple motors of MyoF/Va chimera co-purified with mammalian CamΔall bound to a streptavidin conjugated Qdot 655 (green) on skeletal actin stabilized with Alexa 488 phalloidin (magenta). **No events were observed**. Frames acquired at 50 ms intervals, 37°C. 1x playback, Image width 32.5 μm.

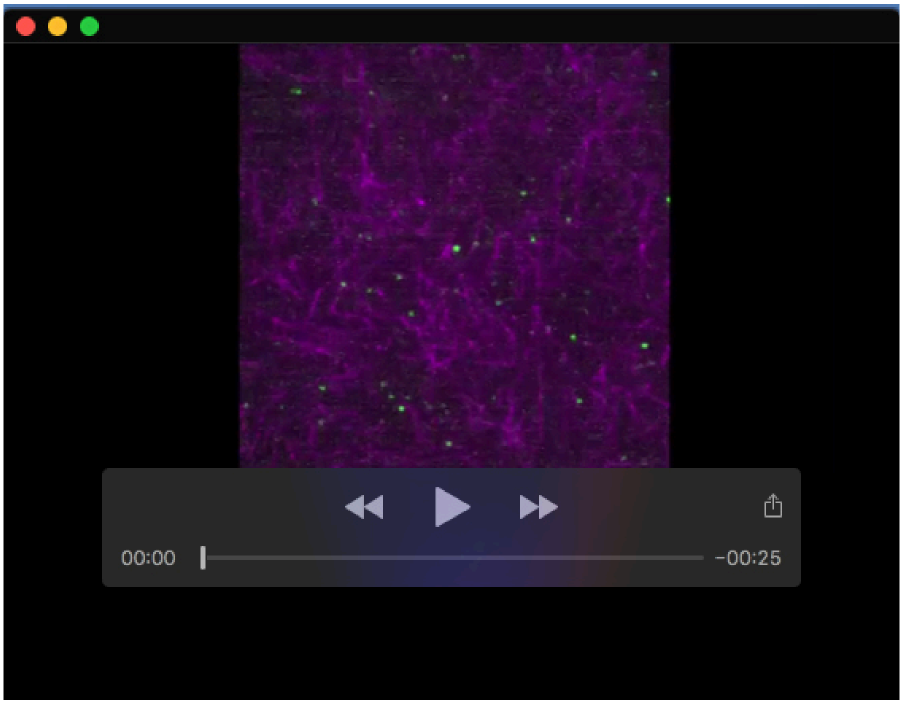

Movie 16. Movement of multiple motors of MyoF/Va chimera co-purified with mammalian CamΔall bound to a streptavidin conjugated Qdot 655 (green) on *Tg*Act1 filaments stabilized with jasplakinolide and imaged with chromobody fused to EmeraldFP (magenta). Frames acquired at 50 ms intervals, 37°C. 3x playback, Image width 13 μm.

Journal of Cell Science • Supplementary information

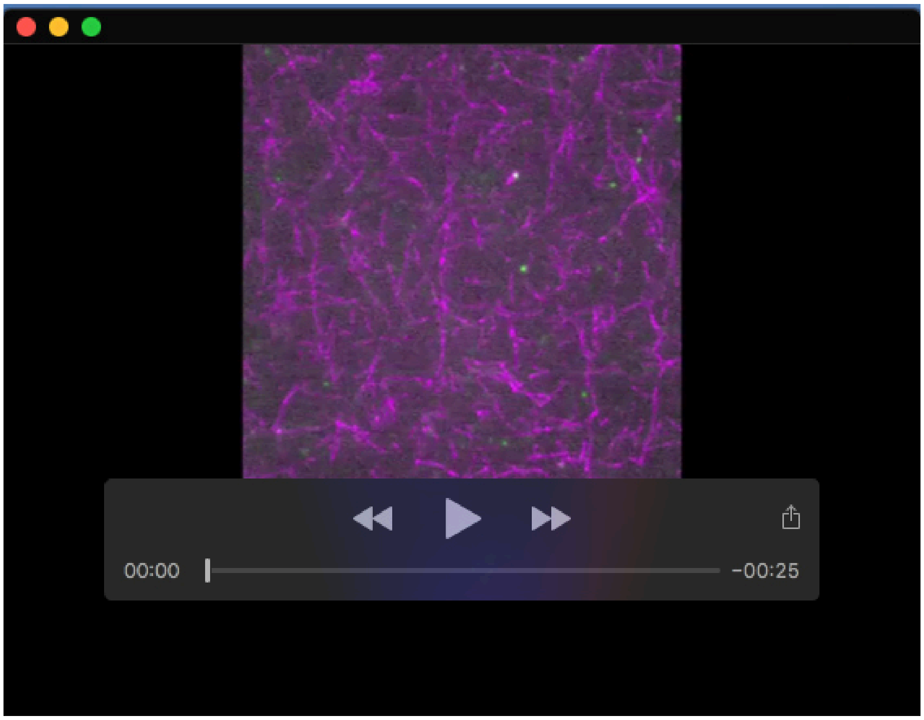

Movie 17. Single molecule motility of MyoF/Va chimera co-purified with mammalian CamΔall bound to a streptavidin conjugated Qdot 655 (green) on *Tg*Act1 filaments stabilized with jasplakinolide and imaged with chromobody fused to EmeraldFP (magenta). **No events were observed.** Frames acquired at 50 ms intervals, 37°C. 3x playback, Image width 32.5 µm.

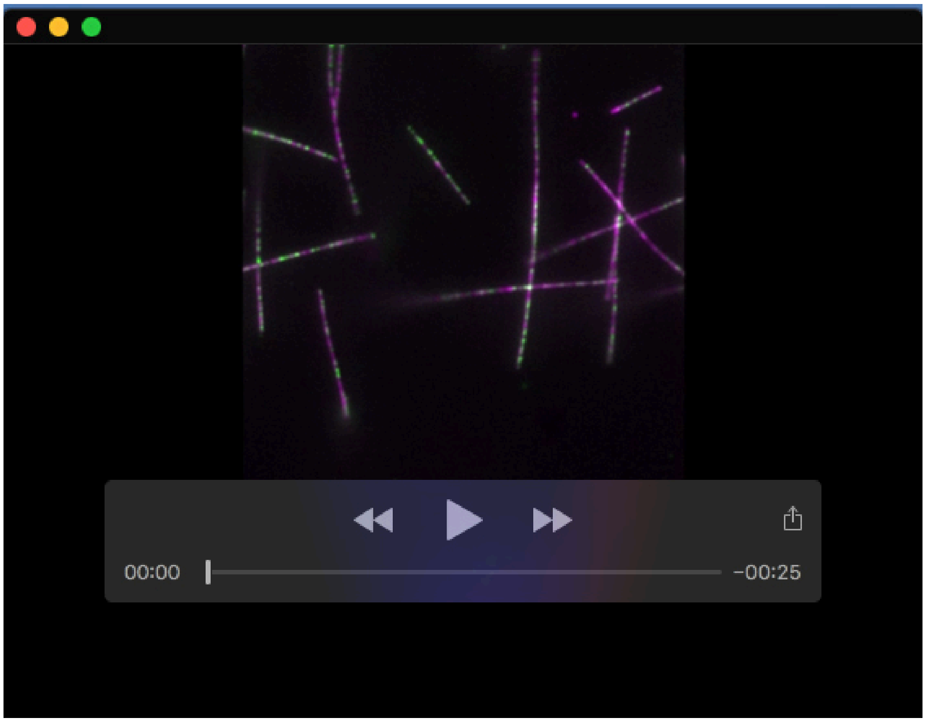

Movie 18. Single molecules of full-length MyoF labeled with Alexa Fluor 647 streptavidin (green) statically associate with Cy5-labeled taxol stabilized microtubules (magenta). Frames acquired at 2 s intervals, 37°C. 16x playback, Image width 32.5 µm.

Journal of Cell Science • Supplementary information

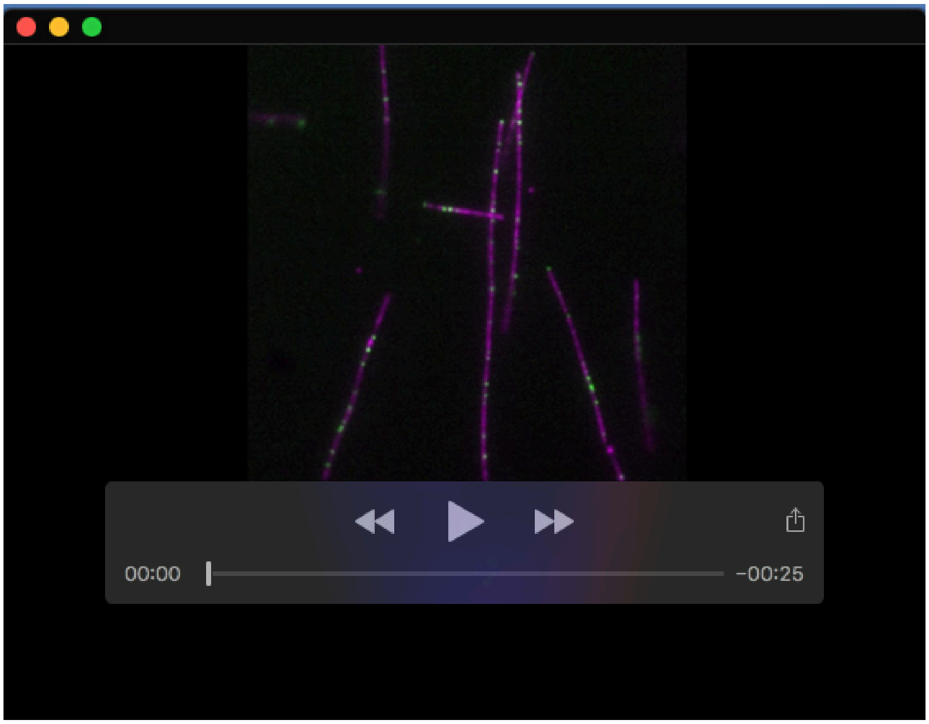

Movie 19. Single molecules of MyoF-Tail labeled with Alexa Fluor 647 streptavidin (green) statically associate with Cy5-labeled taxol stabilized microtubules (magenta). Frames acquired at 2 s intervals, 37°C. 16x playback, Image width 32.5 μm.

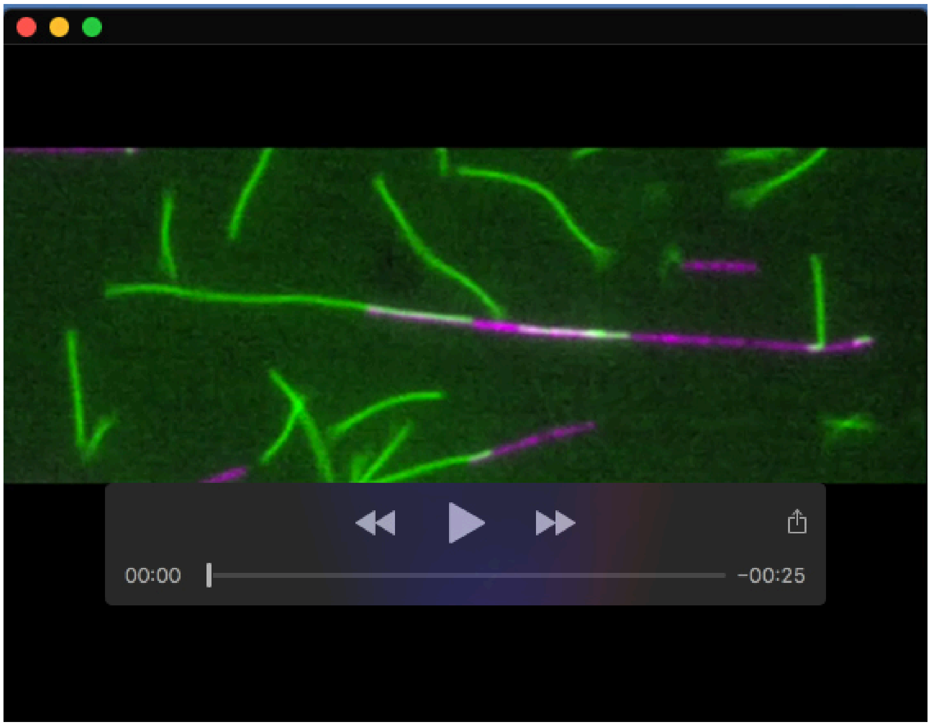

Movie 20. Dynamic actin-microtubule crosslinking assay showing the relative gliding of Alexa Fluor 488 phalloidin stabilized skeletal F-actin (green) by Full-length MyoF (unlabeled) that is bound to a surface immobilized Cy5-labeled microtubule (magenta). Frames acquired at 2 s intervals, 37°C. 40x playback, Image width 36 μm.

Journal of Cell Science • Supplementary information

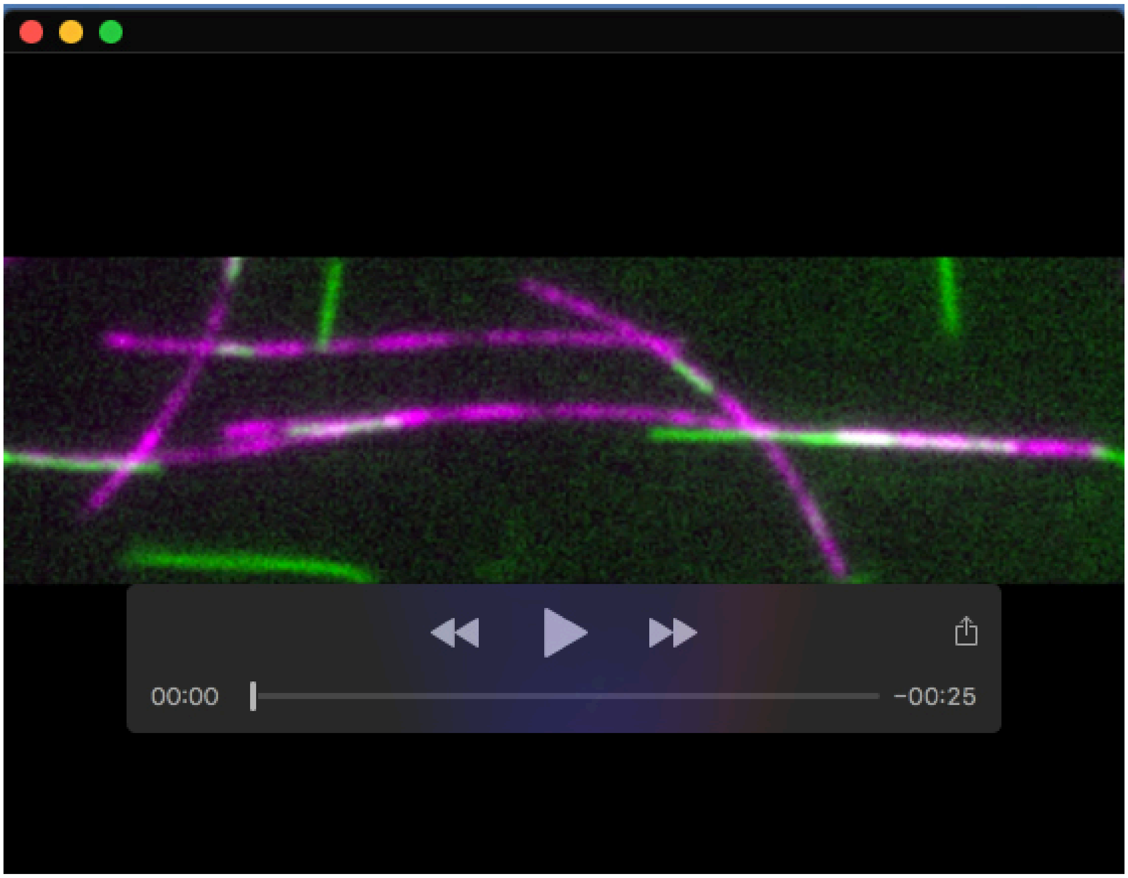

Movie 21. Dynamic actin-microtubule crosslinking assay performed at 150 mM KCl. Shown is the relative gliding of Alexa Fluor 488 phalloidin stabilized skeletal F-actin (green) by Full-length MyoF (unlabeled) that is bound to a surface immobilized Cy5-labeled microtubule (magenta). Frames acquired at 2 s intervals, 37°C. 40x playback, Image width 24 µm.

Journal of Cell Science • Supplementary information
